# Matrix methods in health demography: a new approach to the stochastic analysis of healthy longevity and DALYs

**DOI:** 10.1186/s12963-018-0165-5

**Published:** 2018-06-07

**Authors:** Hal Caswell, Virginia Zarulli

**Affiliations:** 1 Institute for Biodiversity and Ecosystem Dynamics, University of Amsterdam, PO Box 94248, Amsterdam, 1090 GE The Netherlands; 2 Interdisciplinary Center on Research and Education on Population Dynamics (InCent), University of Southern Denmark, Campusvej 55, Odense, DK-5230 Denmark

**Keywords:** Health, Matrix population models, Longevity, Prevalence, Markov chains with rewards, Healthy longevity, Sullivan method

## Abstract

**Background:**

Increases in human longevity have made it critical to distinguish healthy longevity from longevity without regard to health. Current methods focus on expectations of healthy longevity, and are often limited to binary health outcomes (e.g., disabled vs. not disabled). We present a new matrix formulation for the statistics of healthy longevity, based on health prevalence data and Markov chain theory, applicable to any kind of health outcome and which provides variances and higher moments as well as expectations of healthy life.

**Method:**

The model is based on a Markov chain description of the life course coupled with the moments of health outcomes (“rewards”) at each age or stage. As an example, we apply the method to nine European countries using the SHARE survey data on the binary outcome of disability as measured by activities of daily living, and the continuous health outcome of hand grip strength.

**Results:**

We provide analytical formulas for the mean, variance, coefficient of variation, skewness and other statistical properties of healthy longevity. The analysis is applicable to binary, categorical, ordinal, or interval scale health outcomes. The results are easily evaluated in any matrix-oriented software. The SHARE results reveal familiar patterns for the expectation of life and of healthy life: women live longer than men but spend less time in a healthy condition. New results on the variance shows that the standard deviation of remaining healthy life declines with age, but the coefficient of variation is nearly constant. Remaining grip strength years decrease with age more dramatically than healthy years but their variability pattern is similar to the pattern of healthy years. Patterns are similar across nine European countries.

**Conclusions:**

The method extends, in several directions, current calculations of health expectancy (HE) and disability-adjusted life years (DALYs). It applies to both categorical and continuous health outcomes, to combinations of multiple outcomes (e.g., death and disability in the formulation of DALYs) and to age- or stage-classified models. It reveals previously unreported patterns of variation among individuals in the outcomes of healthy longevity.

**Electronic supplementary material:**

The online version of this article (10.1186/s12963-018-0165-5) contains supplementary material, which is available to authorized users.

## Background

Increases in human longevity, driven by improvements in nutrition, public health, medical care, and medical technology have dramatically changed the prospects of future life, especially for the elderly. This has led to the need to distinguish *healthy* longevity from longevity without regard to health, a concept first introduced by [1]. The interest in measuring healthy longevity has spawned a plethora of indices [2–4], including health expectancy (HE), health-adjusted life expectancy (HALE), disability-free life expectancy (DFLE), quality-adjusted life years (QALY), disability-adjusted life years (DALY); see [5] for an overview.

**Fig. 1:**
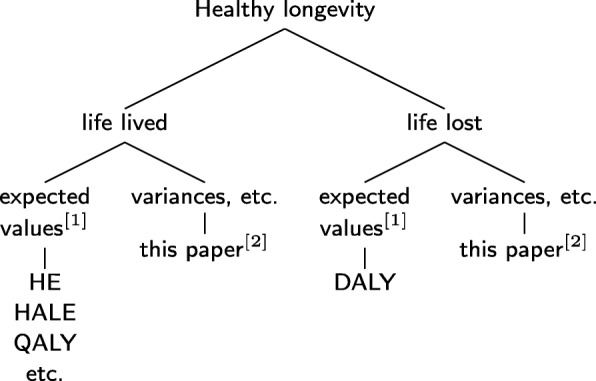
A classification of indices of healthy longevity. Healthy longevity refers to analyses that incorporate mortality and health, and use that information to address questions about length of life. Healthy longevity may focus on life lived in different states of health or life lost due to particular causes of mortality or morbidity. HE = health expectancy. HALE = health adjusted life expectancy. QALY = quality-adjusted life years. DALY = disability-adjusted life years. [1] Analyses routinely report only expected values. [2] In addition to expectations, this method also provides the variance, standard deviation, coefficient of variation, and skewness, as well as higher moments if desired

It is not our purpose here to review these measures. We recommend the detailed and extensive treatment by Siegel, who notes that these categories “are not easily distinguished or even mutually exclusive on close examination” ([5],Chap. 8). In this paper, we use the following definitions, as outlined in Fig. 1. We are concerned with a class of health metrics that evaluate what we call *healthy longevity*. This class includes all analyses that combine information on mortality and on health status, with the intent to relate health and length of life. Our usage is close, but not identical, to what Murray et al. [3] call “summary measures of population health,” although their usage leaves out important aspects.

Measures of healthy longevity can be divided into those that measure the amount or quality of life *lived* in various health states and those that measure the amount or quality of life *lost* due to particular sources of mortality and morbidity. Murray et al. [3] attempted to capture this difference by speaking of “health expectancies” and “health gaps,” but their terminology unfortunately leaves no room for consideration of variances as well as expectations. As shown in Fig. 1, one of our goals is to provide a framework that includes the variance and higher moments of both types of healthy longevity measures.

Healthy longevity by no means exhausts the spectrum of summary measures of population health. For example, some studies describe population health based on age-standardized measures of cause-specific mortality, or on distributions of health indicators such as body mass index or tobacco use; see ([5],Chap. 16) or the book by Keyes and Galea [6]. Even farther removed from healthy longevity are, of course, measures of the reproductive health of populations (rates of pregnancy loss, prevalence of contraceptive use, etc.); see ([5],Chap. 9). None of these important indices satisfy the definition of healthy longevity.

Figure 1 clarifies the demographic and health issues approached by an analysis of healthy longevity: does it consider length of life? does it measure life lived or life lost? is it limited to expected values, or does it also calculate variances and higher moments? The power of the theoretical framework that we present here is that it provides a unified, matrix-based, approach to all of these concepts.

An important distinction is between analyses based on the prevalence of some health condition and those based on incidence of conditions (in the general sense of describing transitions of individuals among health states). Incidence-based calculations have well known advantages (e.g., [3, 7]), but require longitudinal data that is not always available. In this paper, we are concerned with the analysis of prevalence data; we will consider matrix methods for incidence data elsewhere.

These indices all modify the length of life by a system of weights that describe, on some scale, the quality of that life. Each of them is limited in the kinds of conditions considered and the range of insights provided. In this paper, we introduce a matrix approach to the analysis of healthy longevity. Matrix formulations have a long history in demography as an adjunct to life table methods [8–10]. They have several typical advantages. Matrix formulations greatly simplify the notation for calculations that are inherently multidimensional. They yield expressions that are simply and efficiently computable in any matrix-oriented programming language (e.g., MATLAB or R). Perhaps their most important benefit is to facilitate connections with other mathematical results. In our case, a matrix formulation of healthy longevity using finite-state Markov chains with rewards provides access to previously unexamined statistical properties and sensitivity analyses.

Although the literature on healthy longevity is vast [5], in general some important aspects of healthy longevity have been neglected:

- Most prevalence-based analyses are formulated using life tables, and thus apply only to age-classified demography. In this paper, we extend the domain of analysis to include stage-structured and multistate models. The familiar age calculations are a special case.
- Many analyses are restricted to binary health characteristics (e.g., disabled or not disabled). Our approach is equally applicable to binary, nominal, ordinal, and interval measures of health. This greatly extends the possibilities for measuring the elusive concept of “health.”
- Current analyses focus almost exclusively on the *expectation* of healthy life, neglecting other, equally interesting, statistics of longevity. In particular, they ignore inter-individual variability, which is critical to sociology considered as a population science [11]. Our approach yields measures of inter-individual variability, including the variance, skewness, and other statistics. Such process variance must not be confused with the analysis of uncertainty due to parameter estimation error.

The models underlying healthy longevity calculations differ depending on whether they rely on prevalence data (the proportion of a population experiencing a given health condition at a given age), or on incidence data (the rates of transition among health states at a given age). In this paper, we consider the common case of prevalence data, and defer consideration of incidence models to a subsequent paper.

The most commonly used approach to incorporating prevalence data into health longevity is the so-called Sullivan method [12, 13]. It is based on age-specific prevalence of a binary condition (e.g., disabled vs. non-disabled). If the prevalence of being in a healthy, non-disabled condition in age class *j* is *v*_*j*_, and the number of years lived in age class *j* is *L*_*j*_, then the Sullivan method writes health expectancy as

1 $$ HE(x) = \sum_{j=x}^{\infty} L_{j} v_{j}  $$

Written this way, the Sullivan method clearly represents the accumulation of something (fractional years in a healthy condition) over the course of a life. In the next section, we replace this calculation with a more general stochastic model based on a Markov chain description of the life course. The Sullivan method is obtained as a special case.

## Methods: a Markov chain model for healthy longevity

### Notation

Matrices are denoted by upper-case bold symbols (e.g., **P**), vectors by lower-case bold symbols (e.g., ***ρ***). Vectors are column vectors by default. The transpose of **P** is **P**^T^. The vector **1** is a vector of ones. The diagonal matrix with the vector **x** on the diagonal and zeros elsewhere is denoted diag (**x**). The expected value is denoted by *E*(·), the variance by *V*(·), the coefficient of variation by *CV*(·) and the skewness by *Sk*(·). The Hadamard, or element-by-element, product of matrices **A** and **B** is denoted by **A**∘**B**. Transition matrices of Markov chains are written in column-to-row orientation, and hence are column-stochastic. Table 1 summarizes the variables used in the calculations to follow.

**Table 1:** A summary of mathematical notation for the variables used in definition and calculation of healthy longevity using Markov chains with rewards

Symbol	Definition	Dimension
**P**	Markov chain transition matrix	(*ω*+*a*)×(*ω*+*a*)
**U**	Survival and age transition matrix	*ω*×*ω*
**M**	Mortality matrix	*a*×*ω*
**R**_*i*_	Matrix of *i*th moments of rewards	(*ω*+*a*)×(*ω*+*a*)
***ρ***_*i*_	Vector of *i*th moments of lifetime accumulated reward	(*ω*+*a*)×1
$\tilde {\boldsymbol {\rho }}_{i}$	As ***ρ***_*i*_, but for living states	*ω*×1
**Z**	0-1 matrix to select living states	*ω*×(*ω*+*a*)
**N**	Fundamental matrix	*ω*×*ω*
***η***_1_	Vector of *i*th moments of remaining longevity	*ω*×1
*ω*	Number of age classes	scalar
*a*	Number of absorbing (dead) states	scalar

An analysis of healthy longevity includes three components: a demographic model to describe the life course, including mortality risks as a function of age (or some other individual state variable), a specification of the health outcomes to be considered, and an analytical machinery to calculate the resulting statistical properties of healthy longevity:

Here, the life course is described as a discrete-time absorbing Markov chain (see, e.g. [9, 14–16]). The transient states, which we number 1,…,*ω*, correspond to living stages (usually age classes). The absorbing states, which we number 1,…,*α*, correspond to death. Cases with more than one absorbing state, where *α*>1, occur when death is classified by cause of death, age at death, etc. If the stages are numbered so that the transient states precede the absorbing states, the transition matrix of the Markov chain can be written 
2

The matrix **U**, of dimension *ω*×*ω*, describes transitions among the transient states. For an age-classified model, **U** contains survival probabilities on the subdiagonal and zeros elsewhere; e.g. (for *ω*=4),

3 $$ \mathbf{U} = \left(\begin{array}{cccc} 0 & 0 & 0 & 0 \\ p_{1} & 0 & 0 & 0 \\ 0 & p_{2} & 0 & 0 \\ 0 & 0 & p_{3} & \left[p_{4}\right] \\ \end{array}\right),  $$

The entry in the lower right corner is optional; including it makes the last age class open-ended, with an age-invariant survival probability *p*_*ω*_. We will assume throughout that the dominant eigenvalue of **U** is less than 1, so that an individual beginning in any transient state will eventually be absorbed (i.e., will eventually die) with probability 1. Although we will focus on age-classified models, the method applies equally to life cycles classified by stages (e.g., employment status, marital status) or to multistate models combining stages and age classes. Many results familiar from life table analyses can be extended using this framework. For example, the matrix **U** provides all the moments of longevity [10, 15, 17], measures of life disparity [18, 19], and analyses of frailty [20].

The matrix **M** contains probabilities of death; *m*_*ij*_ is the probability that an individual in age class *i* makes a transition into absorbing state *j*. In the simplest case, **M** has only a single row, corresponding to death. However, for DALY calculations, **M** will contain two rows, one representing death due to the cause under consideration, and the other death from all other causes. More generally, **M** can contain transitions to absorbing states defined by age at death, stage at death, cause of death, or combinations of such categories.

#### Markov chains with rewards

To develop analyses of healthy longevity, we extend the basic model to a Markov chain with rewards [21–23]. The concept of “reward” provides a very general way to quantify the value or quality of life. Imagine an individual moving through the states (age classes or health states) of a Markov chain. Suppose that, at each time step, the individual collects a reward. It may be positive or negative, and depends on the transition made by the individual. The reward accumulates as long as the individual lives. In our case the reward will be some measure related to health (e.g., the simple case of a reward of one year of healthy life if the individual can perform all activities of daily living; one year of disabled life if one or more activities are limited). The concept is much more general. For example this approach has recently been applied to demographic analyses of lifetime reproductive output, in which the reward at any age or stage is the production of children or other offspring [16, 24, 25]. It has been used to analyze lifetime economic transfers, in which the reward at any age may be income, expenditures, or deficits, a case where both positive and negative rewards occur [26].

The life cycle is described by the absorbing Markov chain transition matrix in Eq. (2). An individual making the transition from state *j* to state *i* collects a reward *r*_*ij*_ which is a random variable with specified moments (all moments here are moments around zero). Transitions include the possibility of remaining in the same state. The moments of the *r*_*ij*_ are placed in a series of reward matrices; the matrix containing the *k*th moments of the *r*_*ij*_ is denoted **R**_*k*_:

4 $$ \mathbf{R}_{k} = \left(\begin{array}{c} E \left[ r_{ij}^{k} \right] \end{array}\right).  $$

Rewards accumulate over time. We assume — and this is important — that the accumulation stops at death. Because the rewards, the pathways through the life course taken by an individual, and the lifetime of the individual are all random variables, so is the lifetime accumulated reward. Our goal is to calculate its statistical properties, including the mean, variance, standard deviation, coefficient of variation, and skewness, in terms of the transition matrix **P** and the moment matrices **R**_*k*_. To this end, define ***ρ***_*k*_ as the vector (dimension (*ω*+*α*)×1) containing the *k*th moments of accumulated rewards as a function of the initial stage of the individual

5 $$ \boldsymbol{\rho}_{k} = \left(\begin{array}{c} E \left[ \rho_{i}^{k} \right] \end{array}\right).  $$

### Notation alert

The subscripts on the vectors ***ρ***_*k*_ and the matrices **R**_*k*_ denote the order of the moments. When referring to the entries of the vector or the matrix, subscripts refer to the location in the matrix and the order of the moments migrates to become a parenthetical superscript. That is, the *i*th entry of ***ρ***_*k*_ is $\rho _{i}^{(k)}$ and the (*i*,*j*) entry of **R**_*k*_ is $r_{ij}^{(k)}$.

A recursive formula for these moments was given by [16], and an exact solution is given in [24, 25]. Because rewards are not collected in the absorbing states (in our context, this is the eminently reasonable assumption that the dead do not accumulate any form of longevity, healthy or otherwise), we need only compute that part of ***ρ*** corresponding to the transient, living states 1,…,*ω*. Denoting this subvector by $\tilde {\boldsymbol {\rho }}_{k}$, of dimension *ω*×1, we write

6 $$ \tilde{\boldsymbol{\rho}}_{k} = \mathbf{Z} \boldsymbol{\rho}  $$

where

7 $$ \mathbf{Z} = \left(\begin{array}{c | c} \mathbf{I}_{\omega \times \omega} & \mathbf{0}_{\omega \times \alpha} \end{array}\right).  $$

Then the moments of remaining lifetime rewards, for individuals starting in any of the transient states, are given by the entries of the moment vectors $\tilde {\boldsymbol {\rho }}_{i}$, where

8 $$\begin{array}{@{}rcl@{}} \tilde{\boldsymbol{\rho}}_{1} &=& \mathbf{N}^{\text{\tiny \sf T}} \mathbf{Z} \left(\mathbf{P} \circ \mathbf{R}_{1} \right)^{\text{\tiny \sf T}} 1_{s}  \end{array} $$

9 $$\begin{array}{@{}rcl@{}} \tilde{\boldsymbol{\rho}}_{2} &=& \mathbf{N}^{\text{\tiny \sf T}} \left[ \mathbf{Z} \left(\mathbf{P}\circ\mathbf{R}_{2} \right)^{\text{\tiny \sf T}} \mathbf{1}_{s} + 2 \left(\mathbf{U} \circ \tilde{\mathbf{R}}_{1} \right)^{\text{\tiny \sf T}} \tilde{\boldsymbol{\rho}}_{1} \right]  \end{array} $$

10 $$\begin{array}{@{}rcl@{}} \tilde{\boldsymbol{\rho}}_{3} &=& \mathbf{N}^{\text{\tiny \sf T}} \left[ \mathbf{Z} \left(\mathbf{P} \circ \mathbf{R}_{3} \right)^{\text{\tiny \sf T}} \mathbf{1}_{s} + 3 \left(\mathbf{U} \circ \tilde{\mathbf{R}}_{2} \right)^{\text{\tiny \sf T}} \tilde{\boldsymbol{\rho}}_{1} \right.  \\ && + \left. 3 \left(\mathbf{U} \circ\tilde{\mathbf{R}}_{1} \right)^{\text{\tiny \sf T}} \tilde{\boldsymbol{\rho}}_{2} \right]  \end{array} $$

and, in general,

11 $$\begin{array}{@{}rcl@{}} \tilde{\boldsymbol{\rho}}_{m} &=& \mathbf{N}^{\text{\tiny \sf T}} \mathbf{Z} \left(\mathbf{P} \circ \mathbf{R}_{m} \right)^{\text{\tiny \sf T}}  1_{s}  \\ && + \sum_{k=1}^{m-1} {{m}\choose{k}} \mathbf{N}^{\text{\tiny \sf T}} \left(\mathbf{U} \circ \tilde{\mathbf{R}}_{m-k} \right)^{\text{\tiny \sf T}} \tilde{\boldsymbol{\rho}}_{k} \end{array} $$

where

12 $$ \tilde{\mathbf{R}}_{i} = \mathbf{Z} \mathbf{R}_{i} \mathbf{Z}^{\text{\tiny \sf T}}  $$

is the *ω*×*ω* submatrix of **R**_*i*_ corresponding to the transient states. The matrix **N** is the fundamental matrix of the absorbing Markov chain,

13 $$ \mathbf{N} = \left(\mathbf{I}_{\omega} - \mathbf{U} \right)^{-1},  $$

the (*i*,*j*) entry of which is the mean amount of time spent in state *i* by an individual starting in stage *j*. Proofs of (8)–(11) are given in ([25], Theorem 1).

The entries of the first moment vector ***ρ***_1_ give the mean healthy longevity for individuals of each age or stage, where “healthy longevity” is measured by the lifetime accumulated health rewards, for individuals of each age. Depending on the health outcome of interest, this accumulation might be in units of years of life without disability, years of life weighted by some quantitative measure of health, or years of life lost to mortality and morbidity. The variance, standard deviation, coefficient of variation, and skewness of healthy longevity are calculated from the moment vectors, as

14 $$\begin{array}{@{}rcl@{}} V \left(\boldsymbol{\rho} \right) &=& \boldsymbol{\rho}_{2} - \boldsymbol{\rho}_{1} \circ \boldsymbol{\rho}_{1}  \end{array} $$

15 $$\begin{array}{@{}rcl@{}} SD \left(\boldsymbol{\rho} \right) &=& \sqrt{V \left(\boldsymbol{\rho} \right)} \end{array} $$

16 $$\begin{array}{@{}rcl@{}} CV \left(\boldsymbol{\rho} \right) &=& \text{diag} \left(\boldsymbol{\rho}_{1} \right)^{-1} SD \left(\boldsymbol{\rho} \right) \end{array} $$

17 $$\begin{array}{@{}rcl@{}} Sk \left(\boldsymbol{\rho} \right) &=& \text{diag} \left[ V (\boldsymbol{\rho}) \right]^{-3/2} \; \left(\boldsymbol{\rho}_{3} -3 \boldsymbol{\rho}_{1} \circ \boldsymbol{\rho}_{2} \right.  \\ && \left. + 2 \boldsymbol{\rho}_{1} \circ \boldsymbol{\rho}_{1}\circ \boldsymbol{\rho}_{1} \rule{0in}{2ex} \right). \end{array} $$

The skewness, which is dimensionless, measures the symmetry of the distribution of healthy life. Positive skewness implies a long tail of positive values, and vice versa.

The mean vector $\tilde {\boldsymbol {\rho }}_{1}$ gives exactly what the Sullivan method provides, in the special cases to which it applies: the healthy life expectancy. But with negligible additional effort, $\tilde {\boldsymbol {\rho }}_{2}$ and $\tilde {\boldsymbol {\rho }}_{3}$, from Eqs. (9) and (10), quantify the variation among individuals implied by the joint stochastic action of the transition matrix **U** and the reward moment matrices **R**_1_, **R**_2_, and **R**_3_. In the next section we turn to the calculation of these reward matrices for various indices of health and various kinds of data.

## Quantifying health rewards

Health outcomes may be measured on nominal (e.g., disabled or not disabled), ordinal (e.g., mild, moderate, or severe symptoms), or interval (e.g., numerical scores on physical or mental tests, such as blood pressure, BMI, or grip strength) scales. The Markov chain with reward framework can accommodate any of these measurement scales in the construction of the reward matrices **R**_*k*_. We consider some of the most common cases here; the construction of reward matrices is discussed in detail in [25].

### Binary outcomes: health from prevalence

Let *v*_*i*_ denote the prevalence, in age class *i*, of the health state of interest. This could be the prevalence of a disability or its complement, the prevalence of being disability free. Then

18 $${} \text{reward in age class } {i} = \left\{ \begin{array}{ll} 1 & \text{with probability } v_{i} \\ 0 & \text{with probability } 1-v_{i} \end{array} \right.  $$

which is a Bernoulli random variable. The reward matrices are 
19

20 $$ \mathbf{R}_{3} = \mathbf{R}_{2} = \mathbf{R}_{1}.  $$

The last row of the matrices credits individuals who die with 1/2 year of the health condition. The final column of zeros reflects the assumption that no rewards accumulate in the absorbing state.

### Polychotomous nominal health outcomes

Polychotomous health outcomes include more than two conditions, e.g., healthy (H), receiving home care (C), or receiving institutional care (I). Polychotomous outcomes can be combined into groups to create binary outcomes. Given *n* outcomes, there will be ${n \choose k}$ possible groups of *k* outcomes. One might, for example, be interested in longevity in any of the three categories H, C, and I. Or, one might want to analyze the combination H+C corresponding to freedom from institutionalization; the prevalence of this condition is the sum of the prevalences of H and C. Similarly for the combination H+I (no home involvement required) and C+I (not healthy, regardless of whether care is in the home or an institution). The total number of such conditions is

21 $$ \sum_{k=1}^{n-1} {n \choose k}.  $$

### Ordinal scale health outcomes

A health outcome with *n*>2 ordinal measures; e.g., low (L), medium (M), and high (H), can be grouped into binary comparisons in *n*−1 ways that preserve the order (e.g., L vs. M+H and L+M vs. H). Other groupings may also be useful, however. If L, N, and H correspond to low, normal, and high outcomes on some test, then a comparison of L+H vs. N might be relevant. In any case, however, the prevalence of a combination is the sum of the prevalences of the component outcomes.

### Interval scale measures

When nominal and ordinal measures are reduced to binary outcomes, prevalence data is sufficient to determine all the moments of the reward matrices, and thus to calculate the statistics of healthy longevity, however that may be defined. Quantitative measures, on the other hand, do not generally imply a relation between the mean and the other moments, and thus individual data are required. Survey data provide this kind of information, and the reward matrices are created by empirically calculating the moments of the health outcome measure. If *r*_*i*_ denotes the health outcome for age class *i*, then 
22

Caswell and Kluge [26] applied this approach to calculate the moments of lifetime income and expenditures using individual data from the German Income and Expenditure Survey. Our example below will use grip strength measurements from the SHARE survey in European countries, but the principles are identical.

### Variance within and among trajectories

The variance among individuals in lifetime healthy longevity arises from two sources: differences among the trajectories taken by individuals through their lives, and differences in health status at each age or stage within the life cycle. The overall variance in Eq. (14) can be decomposed into these two components.

The variance among trajectories is calculated by eliminating the variance at each age within trajectories, by fixing the rewards at their mean values [24, 25]. Suppose that the prevalence of a condition at some age is, say, 0.3. Then the rewards calculated in (19) and (20) imply that an individual will spend a year with the condition (with probability 0.3) or spend the year without the condition (with probability 0.7). This is obviously a source of variance among individuals.

Under a fixed reward, every individual experiences 30% of the year with the condition and 70% of the year without the condition. In this (hypothetical) situation, the mean reward is the same (0.3 years with the condition), but there is no variance among individuals. Thus the variance calculated over the lifetime is due only to differences in the pathways that individuals follow through their lives. In a purely age-classified model, the pathway is exactly the length of life. In a model that included some other stages (imagine, for example, marital status combined with age), pathways can be more complicated, but the concept is the same. The reward matrix **R**_1_ for fixed rewards is unchanged, but the higher moments become

23 $$\begin{array}{*{20}l} \mathbf{R}_{2} =&\,\, \mathbf{R}_{1} \circ \mathbf{R}_{1} \end{array} $$

24 $$\begin{array}{*{20}l} \mathbf{R}_{3} =&\,\, \mathbf{R}_{1} \circ \mathbf{R}_{1} \circ \mathbf{R}_{1} \end{array} $$

The variance within trajectories is then calculated by subtraction.

## Combining health rewards: disability-adjusted life years

Disability-adjusted life years are a fundamental component of perhaps the most extensive health demography study ever attempted, the Global Burden of Disease (GBD) study [27, 28]. DALYs differ from simple calculations of healthy longevity in several ways. First, they are computed on a cause-specific basis, to calculate the health burden of a specific disease or condition.(In principle, nothing prevents DALYs from being calculated for all causes combined.) Second, DALYs combine mortality and morbidity information to measure health gaps in terms of years lost to both death and disability, and are thus a “health gap” measure in the terminology of Murray et al. [3]. A larger value of DALY implies poorer health and a greater burden of mortality and morbidity due to the cause under consideration.

The two components of DALYs are calculated as follows.

1. Years lost due to mortality. An individual who dies from the cause under consideration loses some number of years of remaining life. The expectation of this loss is the remaining life expectancy at the age of death. The GBD project calculates this from a synthetic life table based on the lowest death rates for each age group among all locations with total population exceeding 5 million ([29], p. 1263). We will generalize this to also account for the variance and other moments of remaining life.
2. Years lost due to disability. The prevalence rates for all the known diseases are multiplied by a disease-specific severity of disability weight that ranges between 0 and 1 based on how disabling the disease is. One year spent in a severely disabling condition, with a weight close to 1, would cause the almost complete loss of that year. One year spent in a lightly disabling condition, whose weight is close to 0, would cause a minor loss in terms of year-time.

Summing the two components and integrating them over age will give global burden of disease of a population, expressed in the total number of years of good health lost due to either death or disability. See [30] for a description of the calculations.

**Fig. 2:**
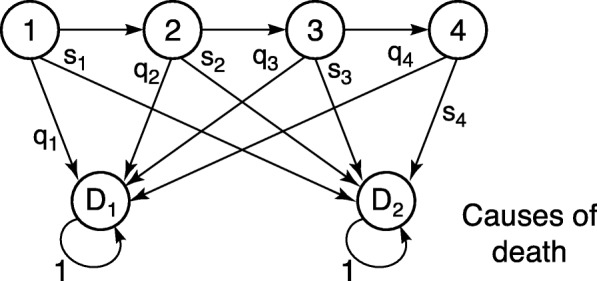
Life cycle. Partial life cycle graph for an age-classified life cycle with with four age classes and two causes of death; *q*_*i*_ and *s*_*i*_ are the probabilities of death due to causes 1 and 2, respectively, in age class *i*


Our matrix formulation of DALY calculations extends the results to include the higher moments and measures of variation among individuals. Figure 2 shows a segment of an age-classified life cycle with two causes of death. Suppose that Cause 1 is the focal cause under investigation; Cause 2 represents all other causes. The transition matrix for individuals following this life cycle graph is 
25

26


where *p*_*j*_ is the survival probability of age class *j*. The mortality matrix **M** contains the probabilities of death from each of the two causes of death.

The reward matrices for this population must include both years of life lost due to mortality and quality of life lost due to disability. Years of life lost are associated with the transition from the living age classes to the first row of the matrix **M**; the reward for this transition is equal to the number of years of life lost due to this death. This number of years is a random variable whose moments we can calculate from a mortality schedule. The choice of this mortality schedule is up to the investigator; but whatever choice is made [29], we will denote the matrix derived from this mortality schedule as **U**_s_.

Let *η*_*j*_ denote the longevity of an individual of age *j* under the standard mortality schedule. The moments of *η*_*j*_ are calculated from the fundamental matrix **N**_s_=(**I**−**U**_s_)^−1^. Let ***η***_*i*_ be the vector containing the *i*th moments of remaining longevity for each age class. The first three of these moment vectors are given by

27 $$\begin{array}{*{20}l} \boldsymbol{\eta}_{1}^{{\text{\tiny \sf T}}} &=  1^{{\text{\tiny \sf T}}} \mathbf{N}_{\mathrm{s}} \end{array} $$

28 $$\begin{array}{*{20}l} \boldsymbol{\eta}_{2}^{{\text{\tiny \sf T}}} &= \boldsymbol{\eta}_{1}^{{\text{\tiny \sf T}}} \left(2 \mathbf{N}_{\mathrm{s}} - \mathbf{I} \right) \end{array} $$

29 $$\begin{array}{*{20}l} \boldsymbol{\eta}_{3}^{{\text{\tiny \sf T}}} &= \boldsymbol{\eta}_{1}^{{\text{\tiny \sf T}}} \left(6 \mathbf{N}_{\mathrm{s}}^{2} - 6 \mathbf{N}_{\mathrm{s}} + \mathbf{I} \right)  \end{array} $$

[15, 17, 31]. These vectors provide the years of life lost due to mortality when incorporated into the entries of the reward matrices **R**_*i*_ that correspond to transitions to absorbing state 1.

Individuals who do not die from cause 1 will lose partial years of life due to disability from cause 1. This loss depends on the prevalence and the severity of disability. Let *v*_*j*_ be the prevalence of disability due to cause 1 in age class *j*, and let *s*_*j*_ be the severity of such disability, where 0≤*s*_*j*_≤1. A severity of 1 would imply that the quality of life under the disability is equivalent to death. The GBD study makes a great effort to estimate the prevalence and severity of disability due to large numbers of causes in countries around the world [28].

The reward, measuring years of life lost due to disability from the specified cause, during the survival transition from age *j* to age *j*+1 is

30 $$ r_{j+1,j} = \left\{ \begin{array}{ll} s_{j} & \text{with probability } v_{j} \\ 0 & \text{with probability } 1-v_{j} \end{array} \right.  $$

Combining the two rewards, the moment matrices are: 
31


32



33


These expressions can be concisely written in matrix notation. Define the *ω*×*ω* matrices **V** and **S** to have the entries of **v** and **s**, respectively, on the subdiagonal and zeros elsewhere, and define the unit vector $\mathbf {e}_{1} = \left (\begin {array}{cc} 1 & 0 \end {array}\right)^{\text {\tiny \sf T}}$with a 1 in the first entry and a 0 in the second entry. Then, 
34


35



36


Given the Markov chain matrix **P** in Eq. (25) and the reward matrices **R**_*i*_, the calculation of the moments of the lifetime accumulated reward proceed as for binary and quantitative measures of health. The results would would provide not only the *expected* DALYs, but also the moments, variances, and skewness of disability-adjusted life years, for individuals of any age class.

Because DALYs are a very sophisticated measure of healthy longevity, it is worth summarizing the data required for their calculation:

1. for the population and condition under investigation, age-specific mortality rates due to that condition, and due to all other causes,
2. for the population and condition under investigation, the age-specific prevalence and severity of disability due to that condition, and
3. for the standard population, the age-specific mortality schedule due to all causes.

## An example: healthy longevity from SHARE

In this section, we present example calculations of healthy longevity in terms of a binary outcome (disabled or not disabled) and a continuous quantitative variable (grip strength). We compute the mean, standard deviation, coefficient of variation, an skewness of longevity and of healthy longevity, and compare the patterns across several countries.

We obtained information on the prevalence of disability from survey data reporting results for individuals, in this case the Survey of Health, Ageing, and Retirement in Europe (SHARE), and combined this with age-specific mortality information from the Human Mortality Database [32].

SHARE is a longitudinal survey containing information on more than 85,000 individuals, aged 50 and over, in 20 European countries. It comprises several waves and ad-hoc modules on specific topics, which make it a very complex database suitable for studying a wide range of research questions. We used the simplified version easySHARE [33], which includes the same number of observations as SHARE but is restricted to a subset of variables covering demographics, household composition, social support and network, childhood conditions, health and health behavior, functional limitation indices, work and money.

From easySHARE we selected data for the most recent available wave (wave 4), corresponding to year 2011, and for the nine countries for which a mortality schedule in the year 2011 was available on the HMD (Germany, Sweden, France, Denmark, Switzerland, Belgium, Czech Republic, Portugal and Estonia). Table 2 reports the sample size by country. We terminated calculations at age 90 to avoid erratic results due to small sample sizes for prevalences at ages beyond 90.

**Table 2:** SHARE wave 4: Sample size of the respondents in the countries selected for the analysis

	Female	Male
Belgium	2937	2363
Czech Republic	3542	2576
Denmark	1240	1036
Estonia	4080	2748
France	3345	2512
Germany	836	736
Portugal	1185	895
Sweden	1057	894
Switzerland	2068	1682

**Disability-free longevity.**

As a binary outcome we defined as healthy individuals with no limitations in any of the five activities of daily living as reported in the SHARE variable *adla*. We defined *v*_*i*_ as the proportion of healthy individuals in age class *i* and calculated reward matrices based on Eqs. (19) and (20). To obtain total longevity, we repeated the calculations, setting *v*_*i*_=1 for all age classes (i.e., counting a full year of life for each year lived).

We calculated the vectors $\tilde {\boldsymbol {\rho }}_{1}$, $\tilde {\boldsymbol {\rho }}_{2}$, and $\tilde {\boldsymbol {\rho }}_{3}$ containing the moments of remaining longevity and healthy longevity, and from those vectors calculated the variance, standard deviation, coefficient of variation, and skewness of longevity according to Eqs. (14)–(17).

**Health as measured by grip strength**

Hand grip strength is an index of overall muscular strength, and has been found to be inversely associated with all-cause mortality, cardiovascular mortality, myocardial infarction, and stroke (e.g., [34]). A standardized measure of grip strength, using a dynamometer, is reported in units of kg in the SHARE variable *maxgrip*. We computed the first three moments of *maxgrip* from the SHARE data, and constructed reward matrices according to Eq. (22). We calculated the vectors $\tilde {\boldsymbol {\rho }}_{1}$, $\tilde {\boldsymbol {\rho }}_{2}$, and $\tilde {\boldsymbol {\rho }}_{3}$ containing the moments of remaining lifetime grip strength, and from those calculated the variance, standard deviation, coefficient of variation, and skewness according to Eqs. (14)–(17).

## Results

The results of the calculations can be displayed and compared in various ways. Table 3 shows the mean, SD, CV, and skewness of longevity and healthy longevity up to age 90. Results are shown for men and women, at ages of 55 and 75 years, in the arbitrarily chosen country of Belgium.

**Table 3:** The mean, standard deviation (SD), coefficient of variation (CV) and skewness (Sk) of longevity and healthy longevity to age 90, for Belgium. Healthy longevity is defined as life spent with no limitations of activities of daily living, obtained from easySHARE data. Mean and SD are in units of years; CV and Sk are dimensionless

		Men			Women	
		55	75		55	75
mean	L	24.5	10.1		27.9	11.8
	HL	20.6	7.6		22.2	7.9
SD	L	9.4	5.1		8.5	5.1
	HL	7.6	3.8		6.4	3.2
CV	L	0.38	0.50		0.31	0.40
	HL	0.37	0.50		0.29	0.41
Sk	L	-0.75	-0.38		-1.34	-0.89
	HL	-0.80	-0.28		-1.35	-0.51

For men, life expectancy is 20% longer than healthy life expectancy at age 55, and 13% longer at age 75. For women the same comparison is 26% and 15%. The SD of longevity is 25–60% larger than the SD of healthy longevity. Because the mean and SD vary together, the CVs of longevity and healthy longevity are almost identical, at 0.3–0.5. The CV for both men and women increases with age. Skewness is negative for both longevity and healthy longevity.

**Fig. 3:**
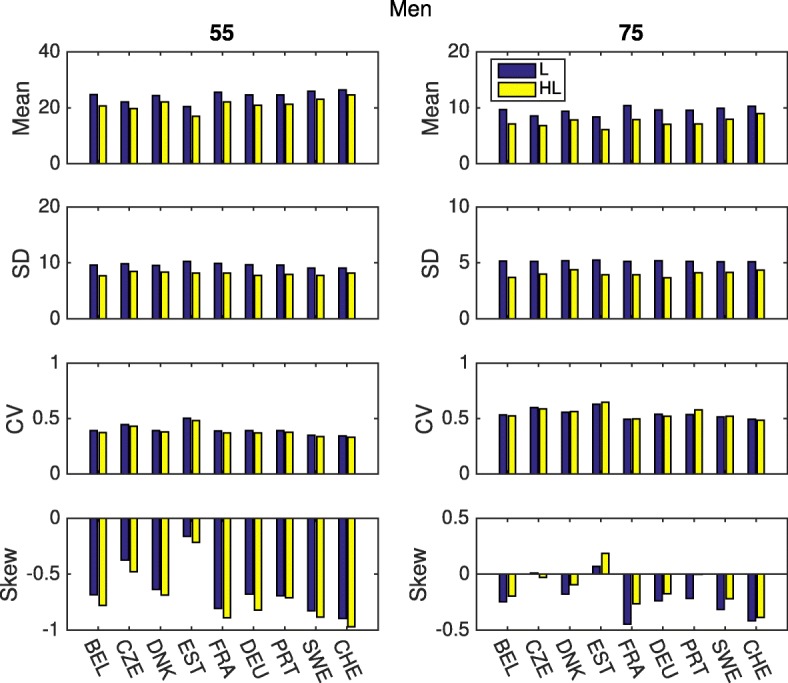
Statistics of longevity and healthy longevity, ADL, men. Mean, Standard Deviation, Coefficient of variation and Skewness up to age 90, for different countries, at ages 55 and 75. Health is defined as the absence of any limitation of activities of daily living

**Fig. 4:**
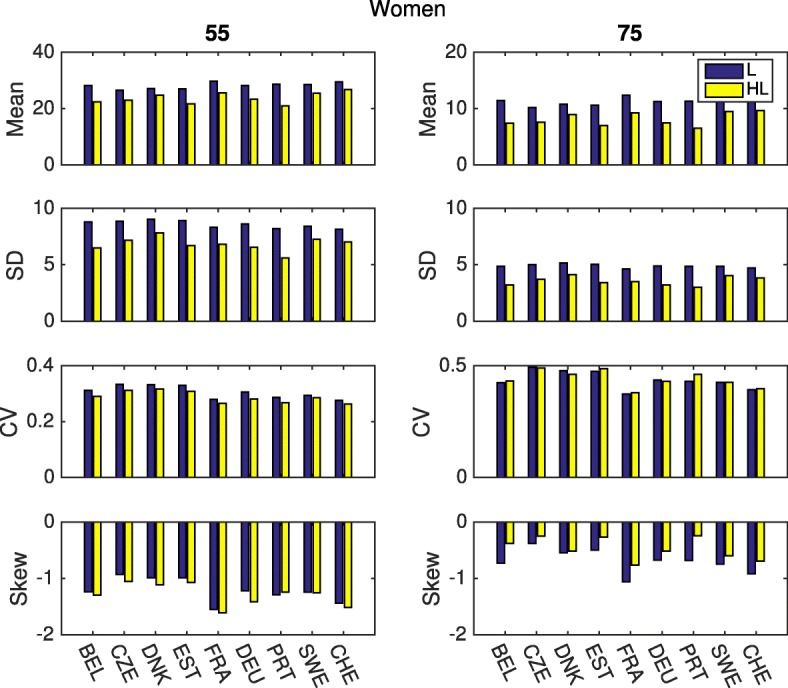
Statistics of longevity and healthy longevity, ADL, women. Mean, Standard Deviation, Coefficient of variation and Skewness up to age 90, for different countries, at ages 55 and 75. Health is defined as the absence of any limitation of activities of daily living

Figures 3 and 4 compare these statistics for all nine countries from the easySHARE dataset. There are no dramatic differences among countries, although small quantitative differences are apparent. As we could expect, women live longer than men but have higher proportion of years with disability. At age 55 they can expect to live about 30 years more in all the analyzed countries, while men have fewer years to live. On the other hand, the sex difference in healthy life expectancy is smaller, indicating that women are likely to spend more years with disability than men. Only in Sweden and Denmark are the number of years with disability similar between the two sexes.

**Fig. 5:**
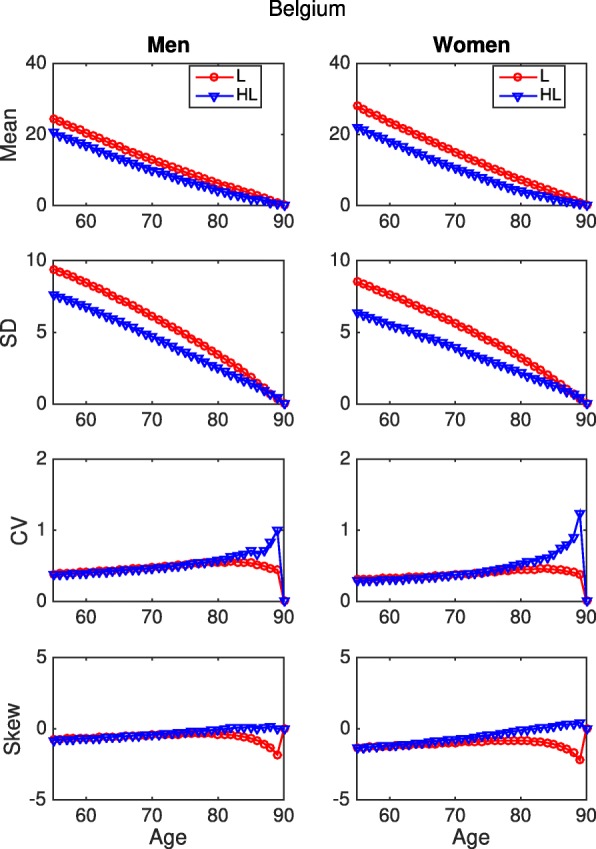
Statistics of remaining longevity and healthy longevity, ADL, Belgium. Mean, Standard Deviation, Coefficient of variation and Skewness up to age 90, based on SHARE data. Health is defined as having no limitation in activities of daily living

Figure 5 shows the entire age schedule of the statistics of remaining healthy longevity for Belgium. The same results for all countries are displayed in the Additional file 1. As they age, both men and women see their inter-individual variation in the expected years of life left decrease from about 9-10 years at the age 55 to about 1-2 years at the end of their life. Moreover, the plots show that the variation in healthy life expectancy is lower than in total life expectancy.

Turning to grip strength as a health outcome, selected statistics at age 55 and 75 for Belgium are shown in Table 4. Unlike disability-free longevity, this measure does not naturally fall into a “healthy” and “non-healthy” outcome. Just as the binary measure based on disability rescales a year of life (to 0 if disabled, to 1 if not disabled), the quantitative measure rescales a year of life to the quality of that life, in this case measured by grip strength. We will refer to lifetime healthy longevity in this case as measured in grip-years (in the same way that person-hours measures work done by some number of persons over some amount of time) Thus, changes in grip-years are a direct measure of the future lifetime prospects for muscular strength and hence revealing about mortality risks.

**Table 4:** The statistics of remaining grip strength years, up to age 90, for Belgium, based on SHARE data. Mean and SD are in units of 10^3^ grip units; CV and Sk are dimensionless

	Men			Women	
	55	75		55	75
mean	1.044	0.366		0.715	0.263
SD	0.370	0.182		0.203	0.104
CV	0.354	0.496		0.283	0.393
Sk	-0.919	-0.368		-1.469	-0.796

**Fig. 6:**
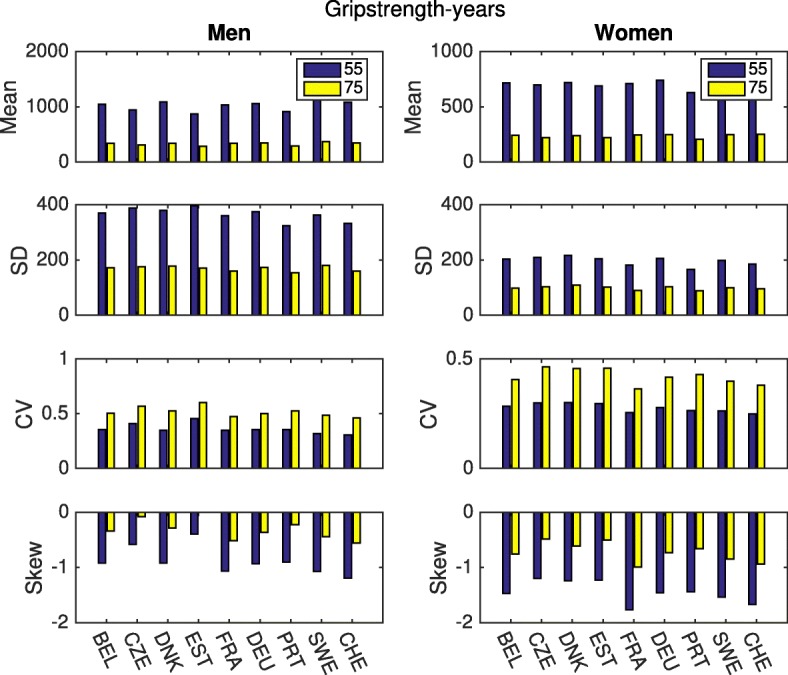
Statistics of longevity and healthy longevity, grip strength. Mean, Standard Deviation, Coefficient of variation and Skewness up to age 90, for men and women, at ages 55 and 75. Health is measured by grip strength; healthy longevity is in units of grip strength-years

A man of 55 can look forward, on average, to 1044 grip-years of life up to age 90. By age 75, this prospect has declined by 65%, to 366 grip-years. A woman of 55 has a grip expectancy of 715 grip-years; by age 75 this has declined by 63%, to 263 grip-years. Variability among individuals, as measured by the CV, is similar to that for disability-free longevity (0.3–0.5). The skewness is negative, and relatively small. Figure 6 compares these statistics across all nine countries. As with disability-free longevity, there are no striking differences, suggesting that these patterns are general for the European countries included in SHARE.

**Fig. 7:**
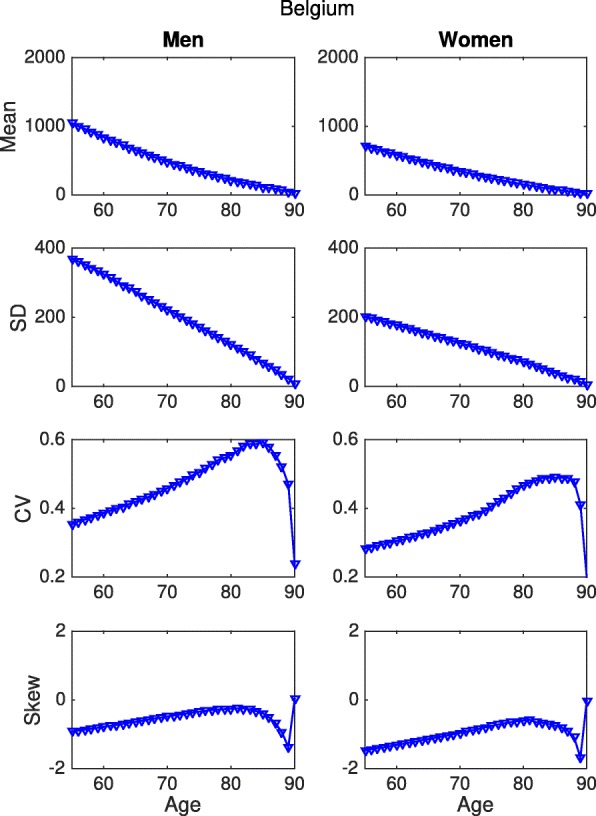
Statistics of remaining longevity and healthy longevity, grip strength, Belgium. Mean, Standard Deviation, Coefficient of variation and Skewness up to age 90, for men and women. Health is measured by grip strength; healthy longevity is in units of grip strength-years

Figure 7 shows the age trajectories of remaining grip strength years for Belgium; again, a gallery of all the countries is presented in the Additional file 1.

## Discussion

We have introduced an approach to healthy longevity that extends current analyses in several directions. It can accommodate any kind of demography, whether based on age, stage, or some combination. It provides information on any kind of health measure, from discrete binary outcomes to continuous interval scale values. It is equally able to analyze “expectancy” or “gap” measures (sensu [3]), and it can combine multiple types of measures (e.g., years lost due to mortality and years lost to disability). It provides a wide range of summary statistics (mean, variance, standard deviation, coefficient of variation, and skewness). And as a additional advantage, the results are easily computable in the form of direct matrix manipulations.

We have emphasized the importance of variances and higher moments (e.g., skewness) of lifetime healthy longevity. These statistics are valuable from both a scientific and an applied perspective. We do not live, work, plan, invest, or make decisions in a world of averages. Any scientific understanding of any process (natural or social) that extends no further than averages is a partial understanding at best, and a misleading one at worst (e.g., [11]). Moreover, in any application with economic implications variance is a source of risk. Attempts to plan investments or allocate resources without considering risk are foolhardy, as is well known in actuarial and financial contexts (see [35]; “An informed discussion of public policy issues, however, requires an analysis of the risks and uncertainties involved. Whether in policies for health or transport, matters monetary or meteorological, in times of war and peace, decisions should reflect a balance of risks. Yet policy debates continue to be permeated by the ‘illusion of certainty.’ ”).

It is important to recognize that stochasticity in the outcome of a process (survival and health, in the present case) is not the same as uncertainty resulting from error in estimation of parameters. Studies of the propagation of uncertainty in order to quantify the consequences of estimation error are based on Monte Carlo sampling from the distributions of parameter estimates (e.g., [36, 37]). An advantage of our method is that uncertainty propagation calculations will be easy to implement because the calculations of healthy longevity are simple and analytical. Note that at least one study has reported that stochasticity in active longevity is much greater than uncertainty due to parameter estimates [38].

Individual-based (or agent-based) microsimulations can also provide information on variance among individuals. However, the prevalence-based calculations addressed here neglect the very kind of individual-level status changes that are the raison d’etre for individual-based simulations. In the present context, an individual-based simulation would be nothing more than a computationally inefficient way to obtain approximations of quantities given exactly by Eqs. (14)–(17).

We focus on prevalence-based analyses here, although such analyses are inherently limited because they do not track the actual movement of individuals among health conditions [3, 7]. Although they require more data than do models based on prevalence, multistate incidence-based models are often applied in health demography [39, 40], medical decision making [41], disease natural history studies [42], and medical follow-up studies [43]. We will present the extension of our methods to incidence-based multistate models in a subsequent paper.

Our two example cases, disability-free longevity and lifetime grip strength, demonstrate the method applied to a binary and a continuous health outcome, respectively. Analysis reveals familiar patterns for the expectation of life and of healthy (disability-free) life: women live longer than men but spend less of that time in a healthy condition. The variability among individuals as measured by the standard deviation declines with age, but the CV, on the order of 0.5, is nearly constant, increasing slightly with age. Remaining grip strength years decrease with age more dramatically than healthy years. Variance also decreases with age, but when standardized relative to the mean, the CV of grip strength years is similar to that for disability-free longevity.

The variance in healthy longevity reported here is the stochastic outcome of the probabilities of survival and of health rewards, applied identically to all individuals as they age. This is *individual stochasticity* in the terminology of [16, 17]. Heterogeneity among individuals certainly exists in the population from which the probabilities are estimated, but once incorporated into the analysis, these differences are discarded and the matrices apply identically to all individuals. To capture the effects of heterogeneity (in frailty, resistance, recovery, health behaviors, etc.) requires a multistate model that incorporates these factors as well as age. Given such a model, the variance in healthy longevity can be decomposed into contributions from stochasticity and heterogeneity (see [20, 44]).

The computational requirements for the implementation of this method are minimal given any programming language that permits matrix operations, including MATLAB and R.

Sensitivity analysis is an important component of any demographic analysis. The goal is to reveal how changes in the parameters affect the results and provide important information on the causal factors determining the results. Sensitivity analyses in health demography are often based on crude numerical manipulations of one a few parameters (e.g., [45, 46]) or by creating some number of scenarios in which entire sets of parameters are manipulated (e.g., [47]).

Because our analysis is formulated in terms of matrix operations, it is amenable to analytical sensitivity calculations based on matrix calculus. The necessary mathematical theory for the sensitivity analysis of Markov chains with rewards has been presented by [25], and will be directly applicable to the statistics of lifetime health. Suppose that ***ξ*** denotes a vector whose entries are age-specific measures of any aspect of lifetime healthy longevity, and let ***θ*** denote a vector containing any parameters that determine mortality and/or health rewards in any way. The mathematical sensitivity analysis of van Daalen and Caswell [25] provides a direct calculation of the derivative matrix

37 $$ {d \boldsymbol{\xi} \over d \boldsymbol{\theta}^{\text{\tiny \sf T}}} = \left(\begin{array}{c} {\partial \xi_{i} \over \partial \theta_j} \end{array}\right)  $$

the (*i*,*j*) entry of which is the derivative of the *i*th entry of ***ξ*** to the *j*th entry in the parameter vector ***θ***. Such sensitivity analyses have now been applied to a variety of demographic models and outputs [18, 19, 48–52]. These calculations provide far more detailed sensitivity information than is provided by constructing scenarios or numerically manipulating parameters. It will be interesting to explore the application of sophisticated sensitivity calculations to the evaluation of potential treatment or intervention strategies.

## Conclusions

We show that healthy longevity can be analyzed by incorporating prevalence of health outcomes into a Markov chain with rewards. The Markov chain defines the transitions and survival between age classes; the rewards specify the moments of the health outcome at each age or stage. Expected healthy longevity is the first moment of lifetime accumulated health. We present analytic formulas for all the moments, extending the results to provide the variance, skewness, and other statistics of healthy life. The analysis applies to binary, nominal, ordinal, or interval scale health outcomes. An analysis of nine European countries based on the SHARE dataset confirm the applicability of the method to both binary (disability-free longevity) and continuous (hand grip strength) measures. Disability-adjusted life years (DALY) is an example of an index that combines two kinds of health outcomes (years lost to death and years lost to disability), and our method readily accommodates this index.

## Additional file

Additional file 1 The second set of figures displays the age schedules of the same statistics of healthy longevity measured by grip strength, for each of the countries in the SHARE dataset. (PDF 384 kb)

## Abbreviations

CV: Coefficient of variation
DALY: Disability-adjusted life years
DFLE: Disability-free life expectancy
GBD: Global Burden of Disease study
HALE: Health-adjusted life expectancy
HE: Health expectancy
HMD: Human Mortality Database
QALY: Quality-adjusted life years
SD: Standard deviation
SHARE: Survey of Health, Ageing, and Disability in Europe
Sk: Skewness
