# Supplementary material for: Matrix methods in health demography: a new approach to the stochastic analysis of healthy longevity and DALYs
Source: Popul Health Metr. 2018 Jun 7;16:8. doi: 10.1186/s12963-018-0165-5 (PMC5992869; doi:10.1186/s12963-018-0165-5)
Supplement: Supplementary file 1 — The second set of figures displays the age schedules of the same statistics of healthy longevity measured by grip strength, for each of the countries in the SHARE dataset. (PDF 384 kb) [file 12963_2018_165_MOESM1_ESM.pdf]

Supplementary material: Matrix methods in health demography: A new approach to the stochastic analysis of healthy longevity and DALYs

Hal Caswell
Virginia Zarulli

April 18, 2018

This document contains two sets of figures. The first set displays the age schedules of the statistics (mean, standard deviation, coefficient of variation, and skewness) of remaining longevity and remaining healthy longevity, up to age 90, for each of the countries in the SHARE dataset. Healthy life is defined by no limitations in activities of daily living.

The second set of figures displays the age schedules of the same statistics of healthy longevity measured by grip strength, for each of the countries in the SHARE dataset.

A Additional material 1 — Statistics of healthy (disability-free) longevity for individual countries

In this Appendix, we display the age schedules of the statistics (mean, standard deviation, coefficient of variation, and skewness) remaining longevity and healthy healthy, up to age 90, for each of the countries in the SHARE dataset. Healthy life is defined by no limitations in activities of daily living.

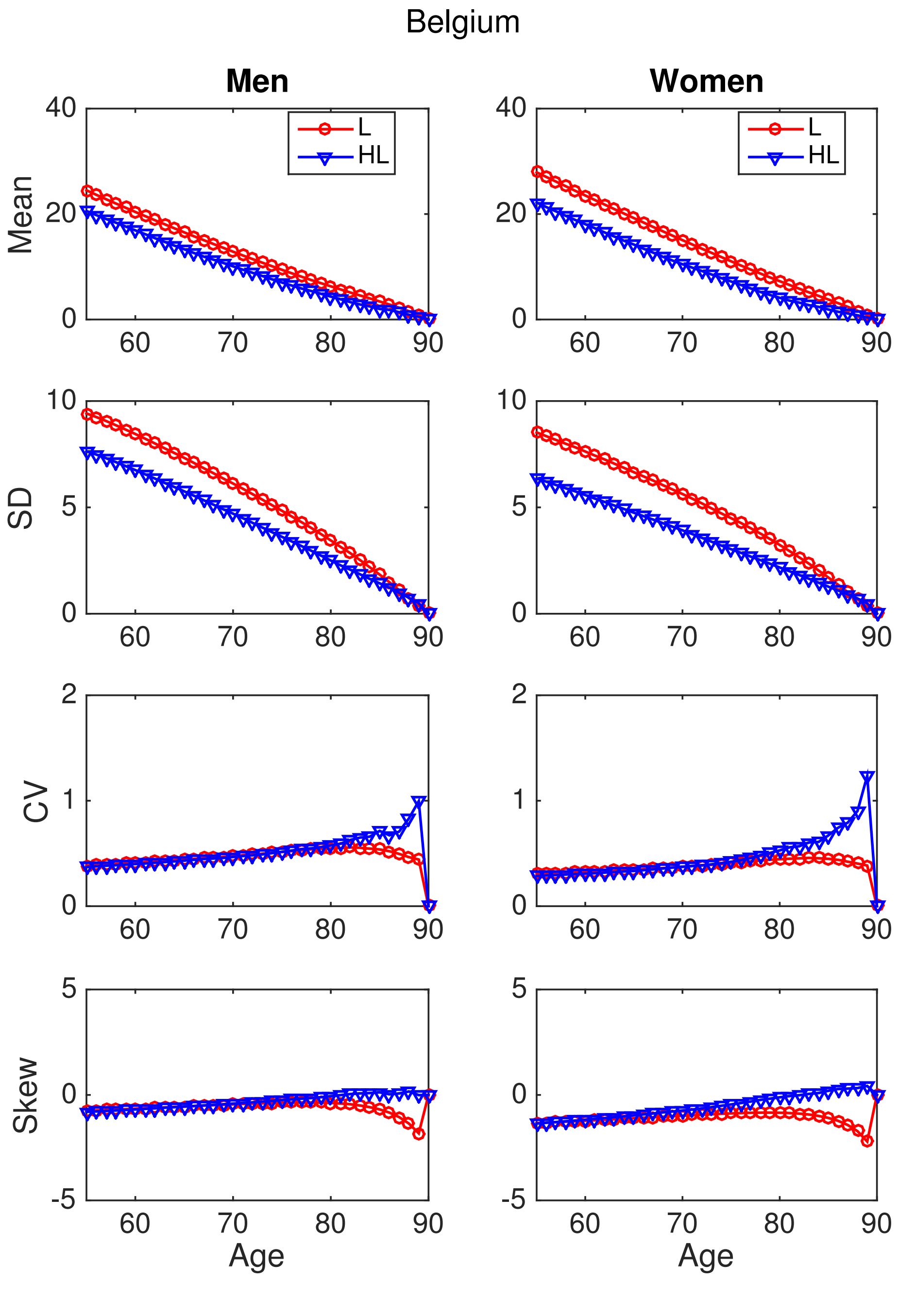

Figure A-1: Mean, standard deviation, coefficient of variation, and skewness for remaining longevity and remaining healthy (disability-free) longevity; Belgium

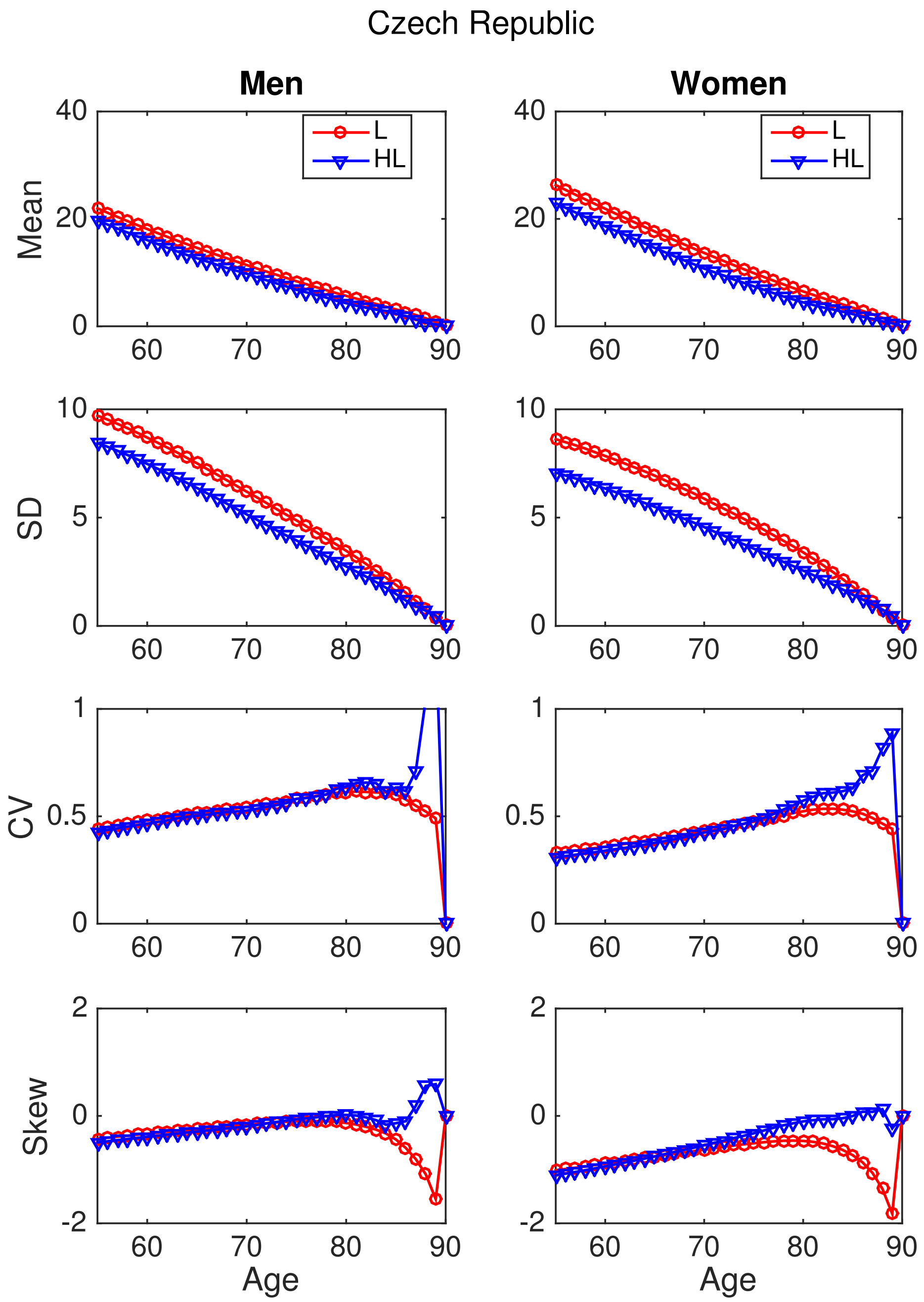

Figure A-2: As in Figure A-1, for Czech Republic

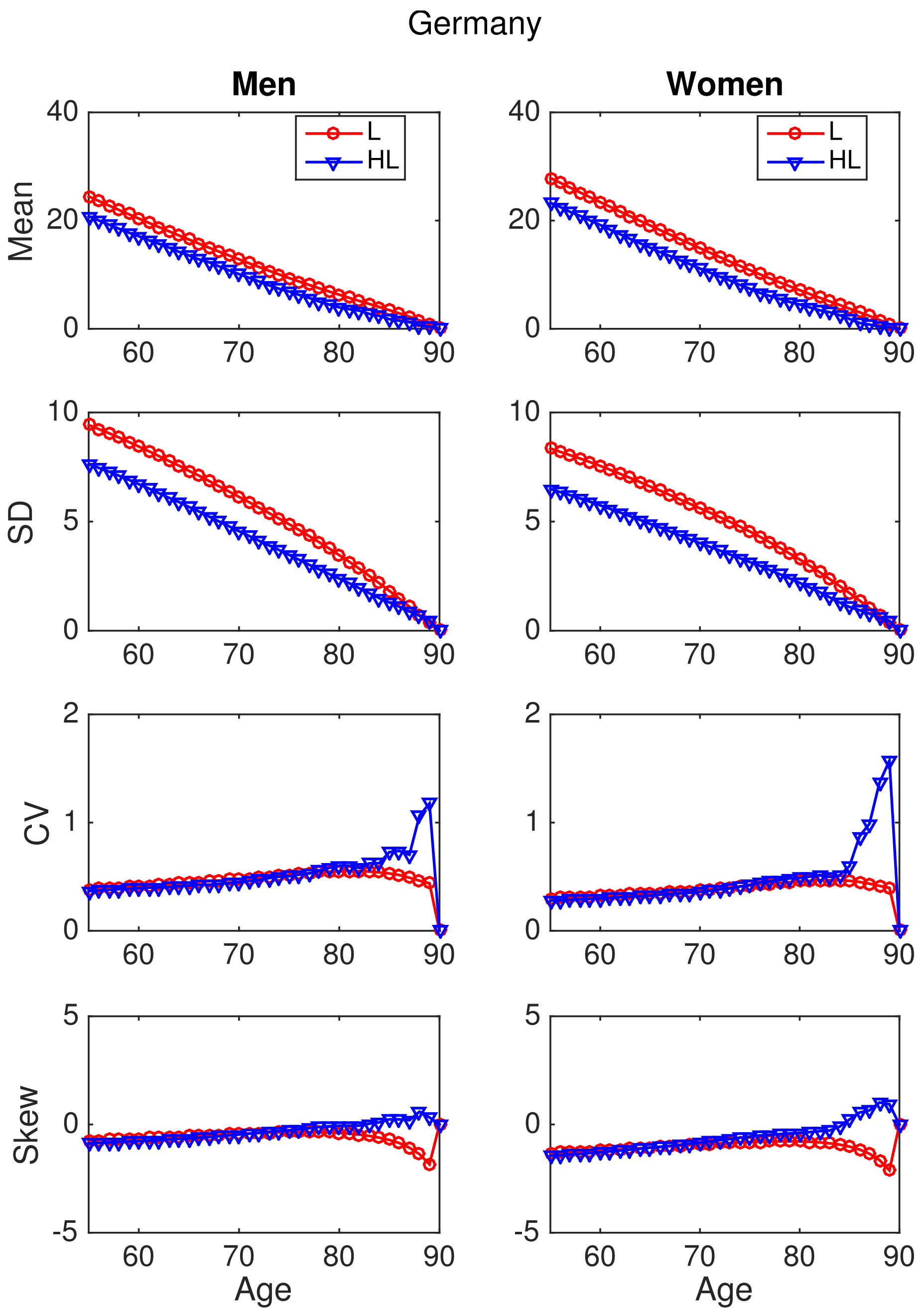

Figure A-3: As in Figure A-1, for Germany

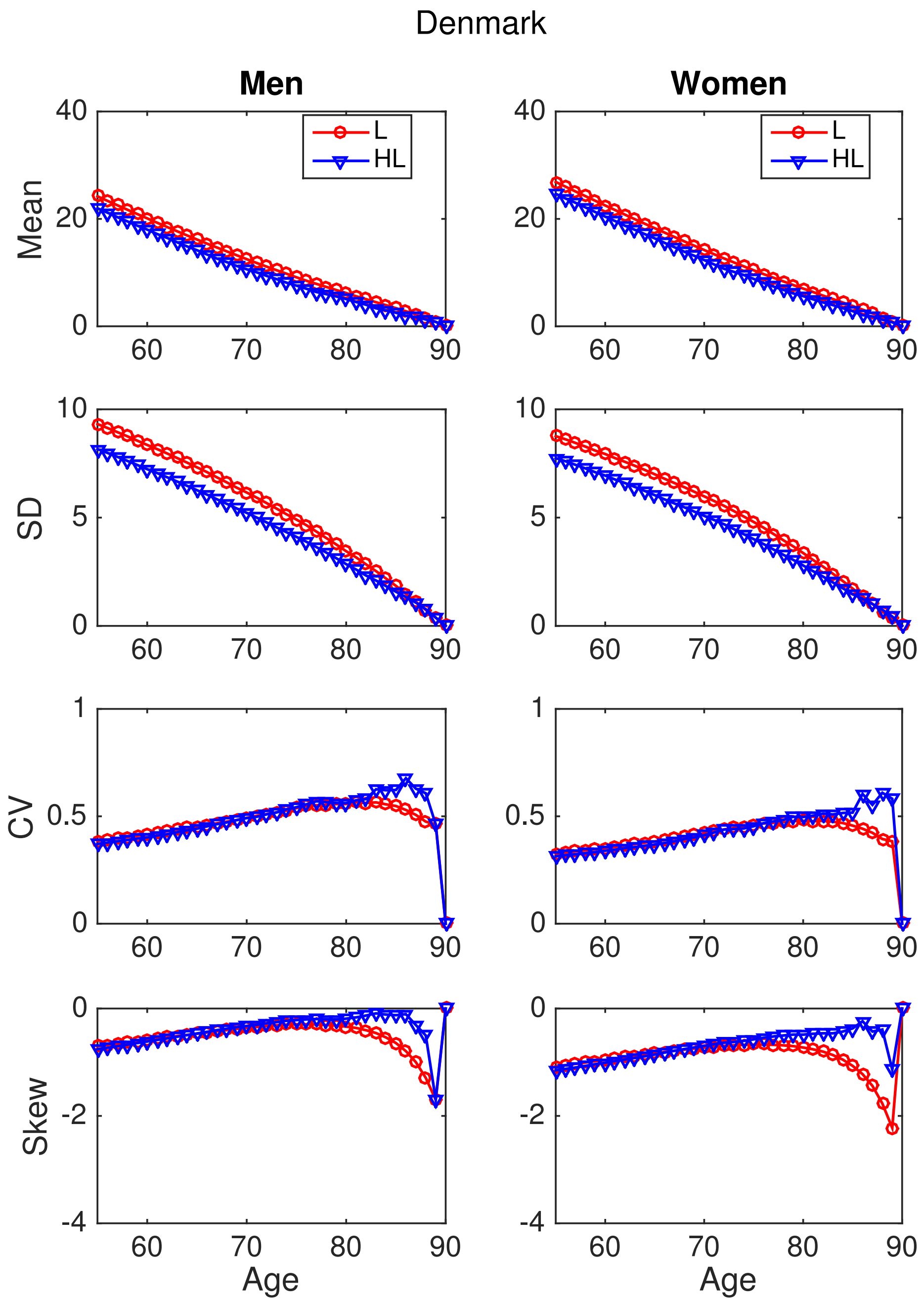

Figure A-4: As in Figure A-1, for Denmark

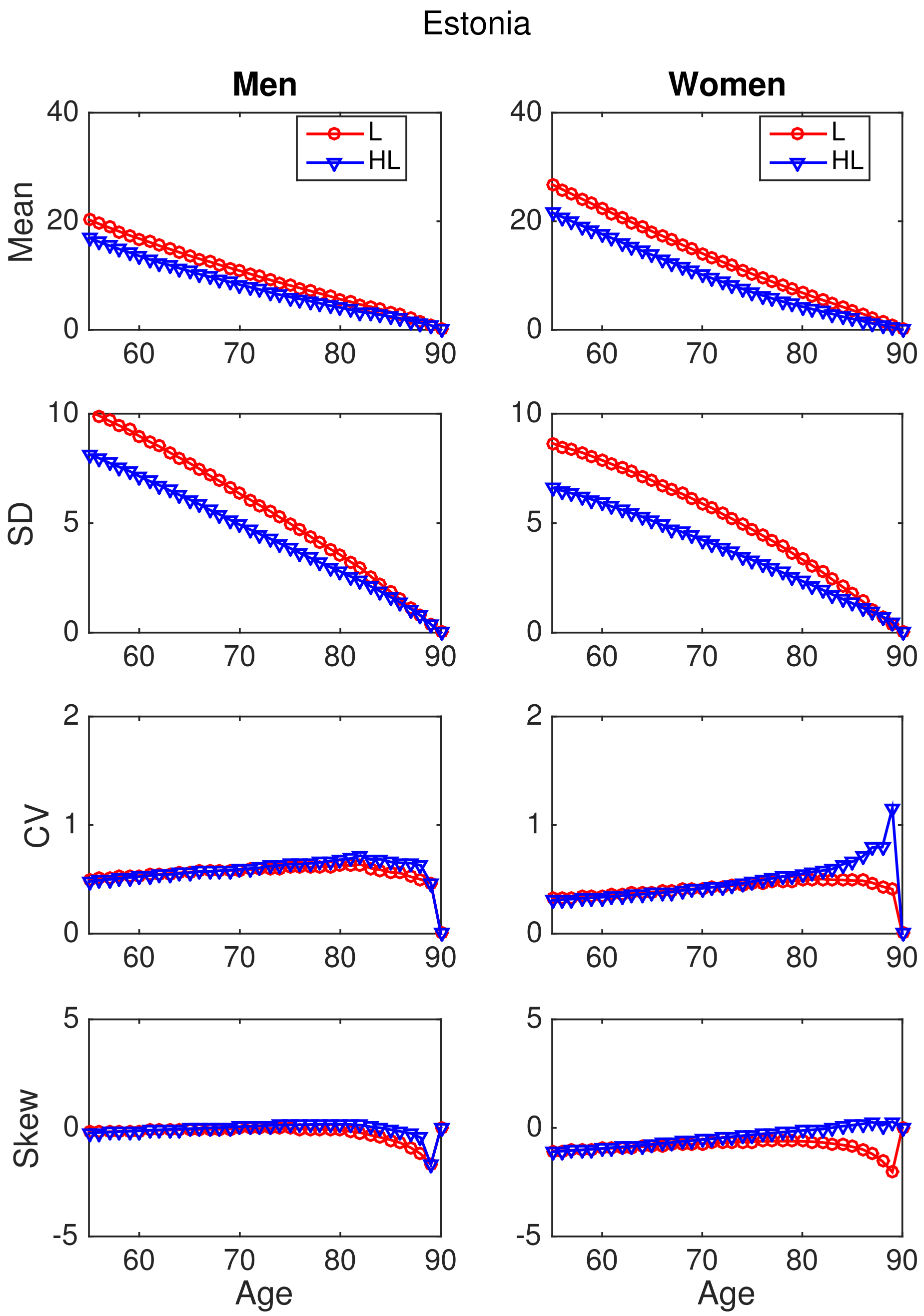

Figure A-5: As in Figure A-1, for Estonia

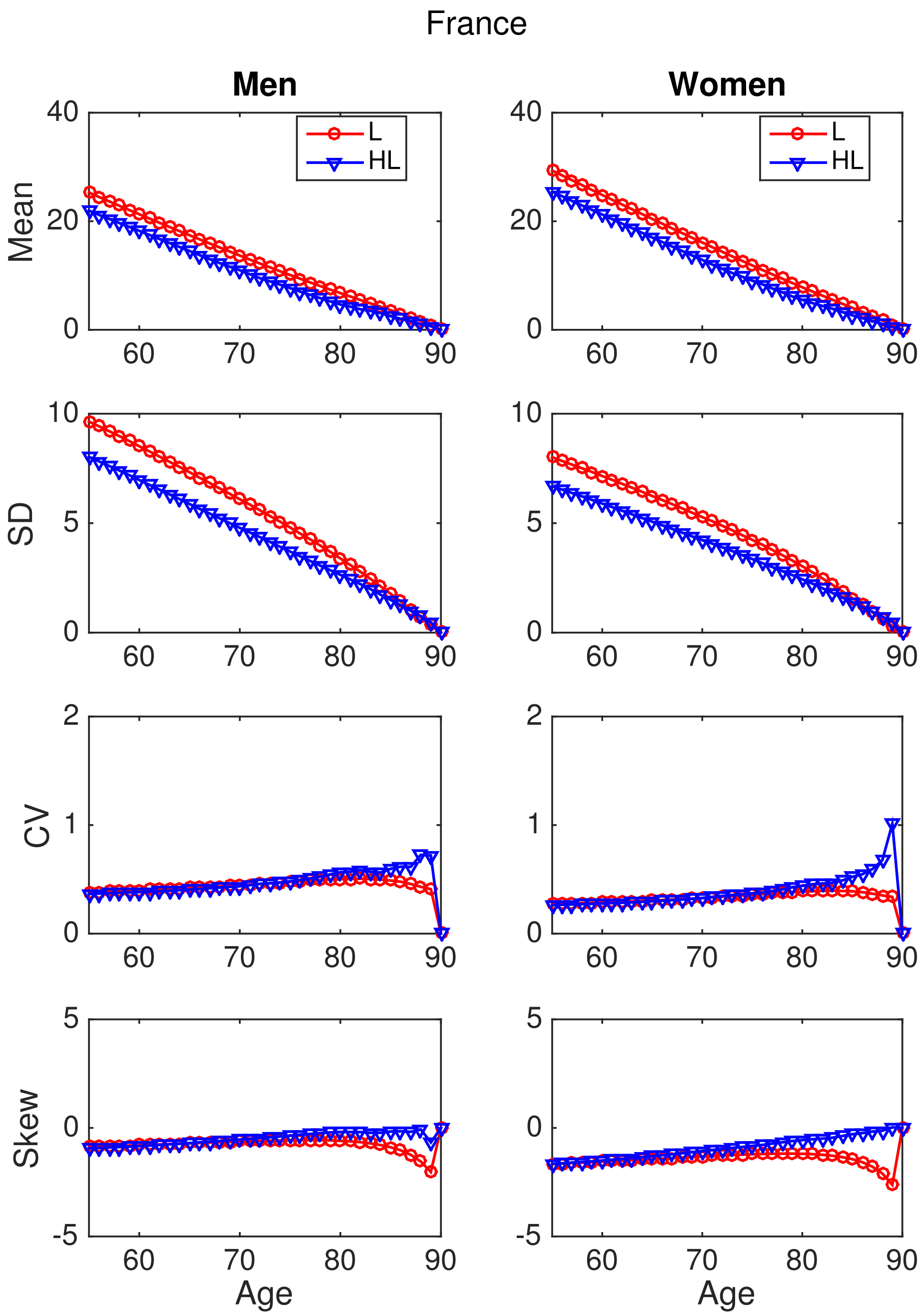

Figure A-6: As in Figure A-1, for France

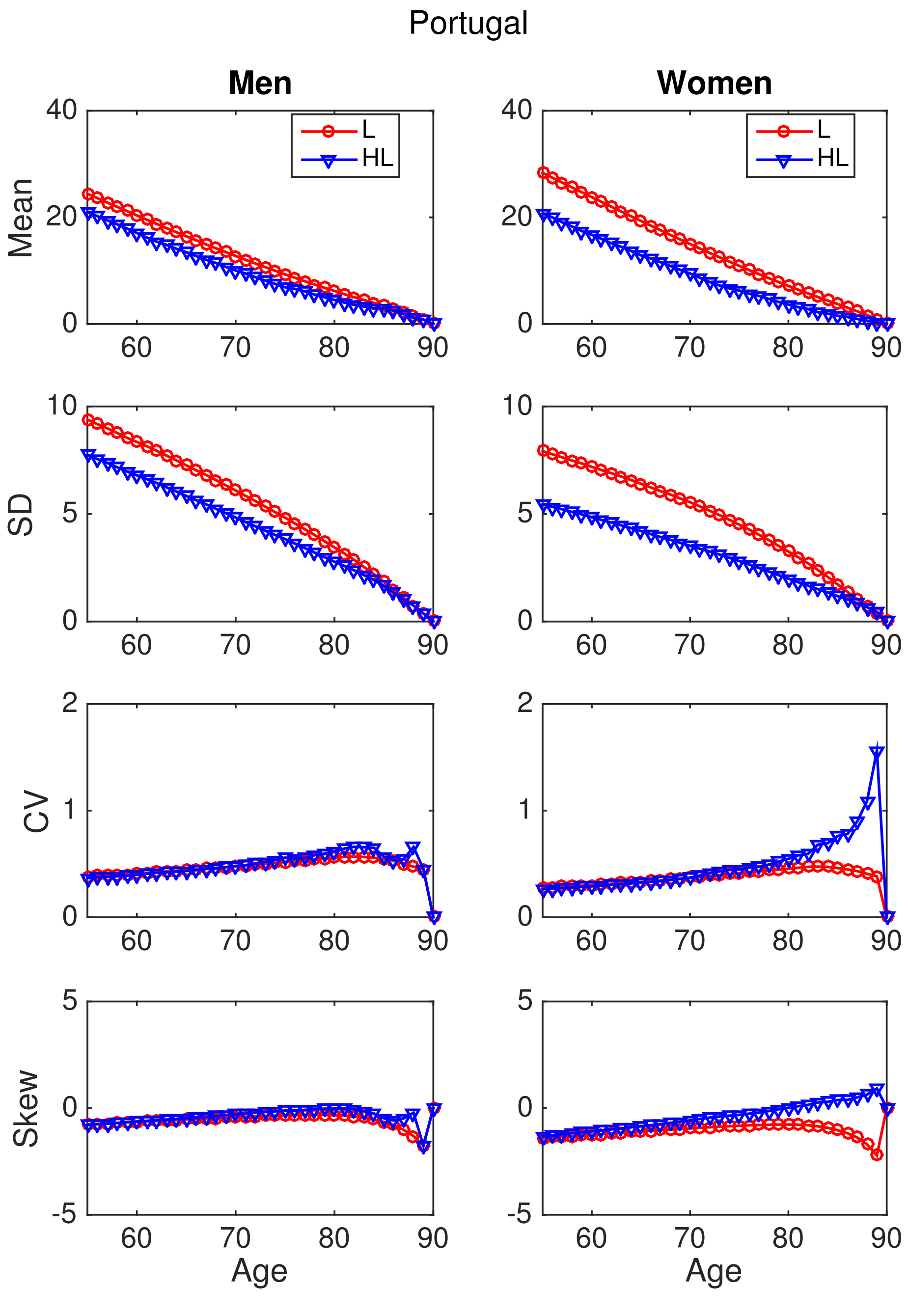

Figure A-7: As in Figure A-1, for Portugal

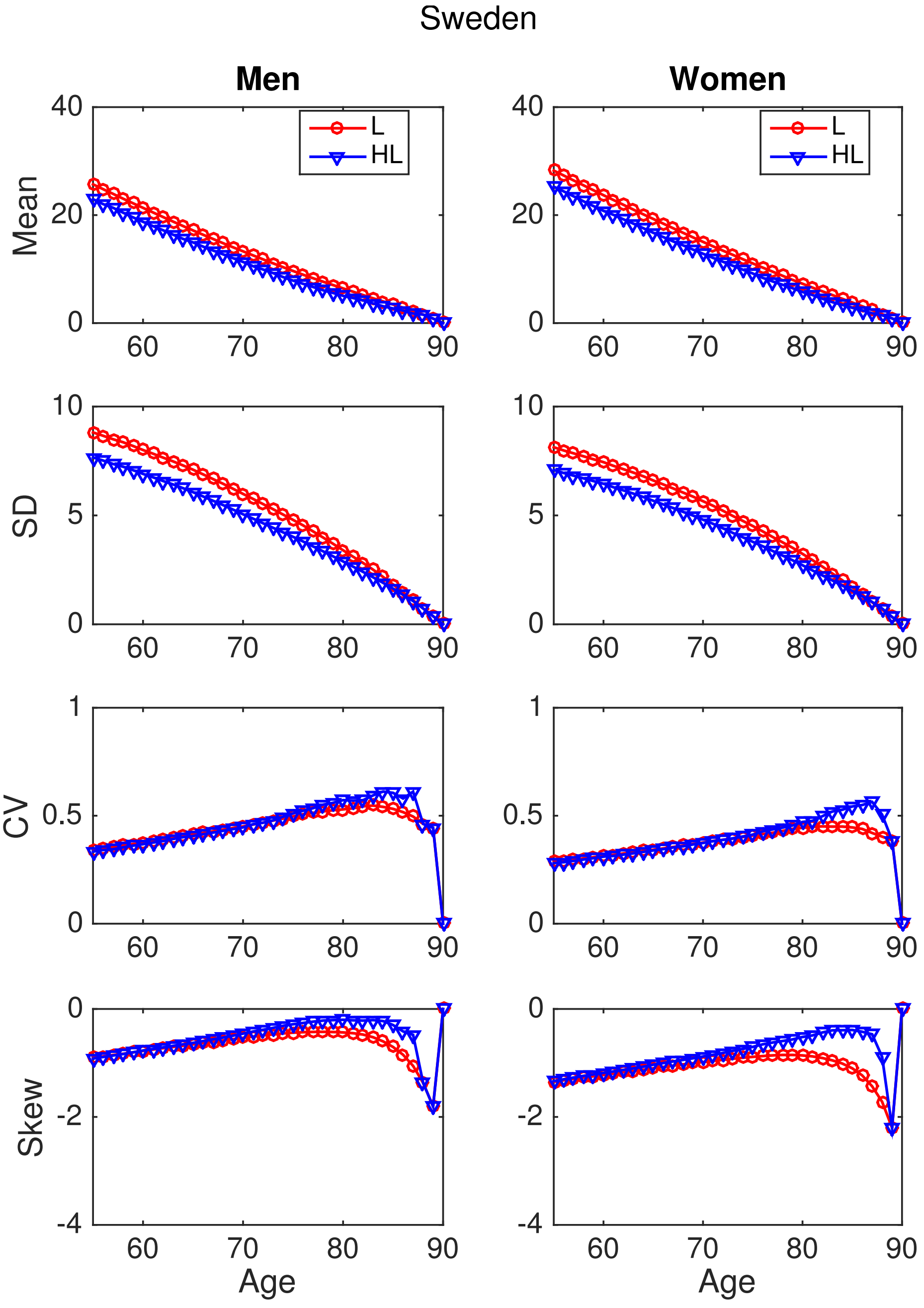

Figure A-8: As in Figure A-1, for Sweden

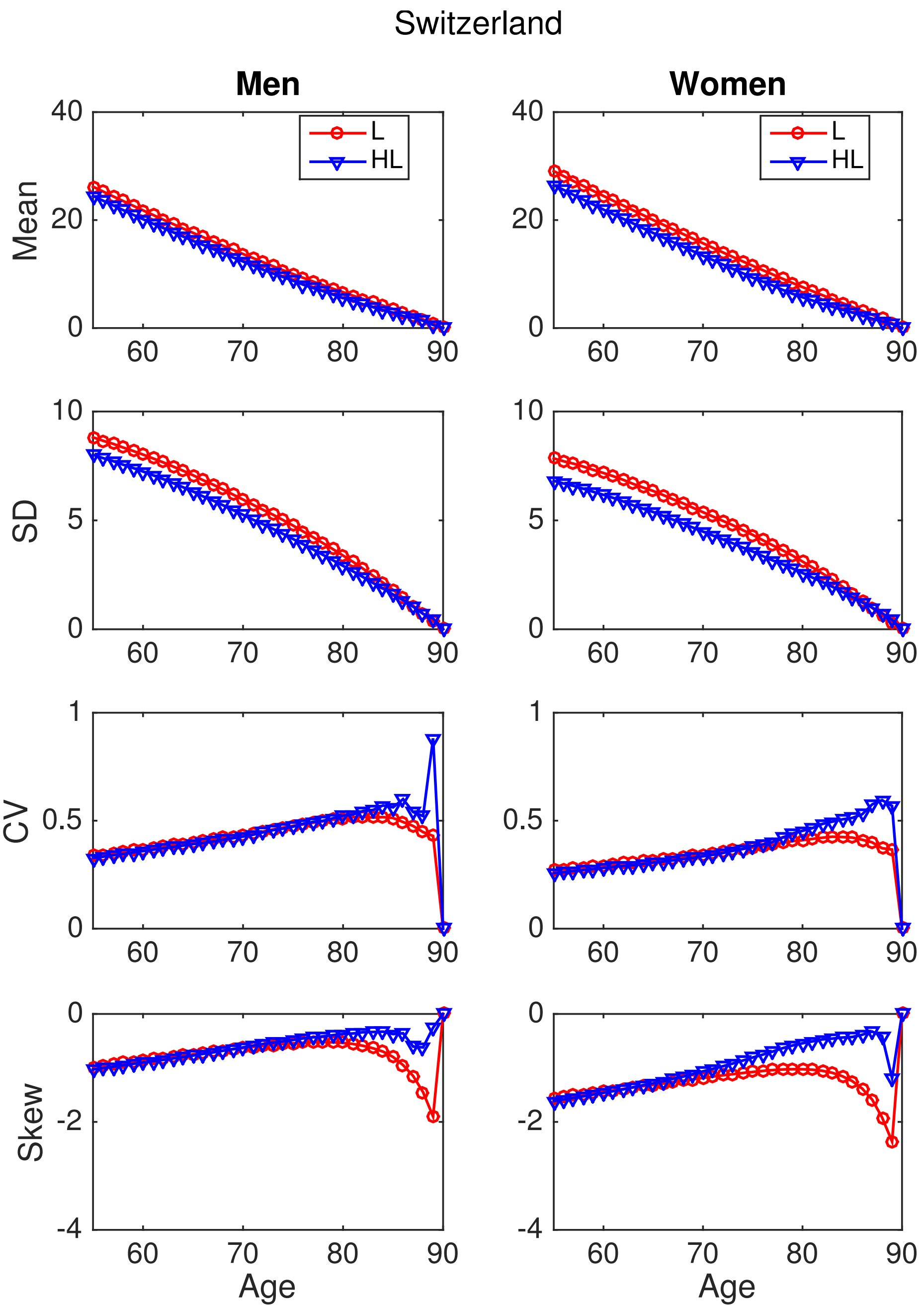

Figure A-9: As in Figure A-1, for Switzerland

B Additional material 2 — Healthy longevity measured by grip strength

In this appendix, we present the age schedules of the statistics (mean, standard deviation, coefficient of variation, and skewness) of remaining grip strength-years for each of the countries in the SHARE dataset.

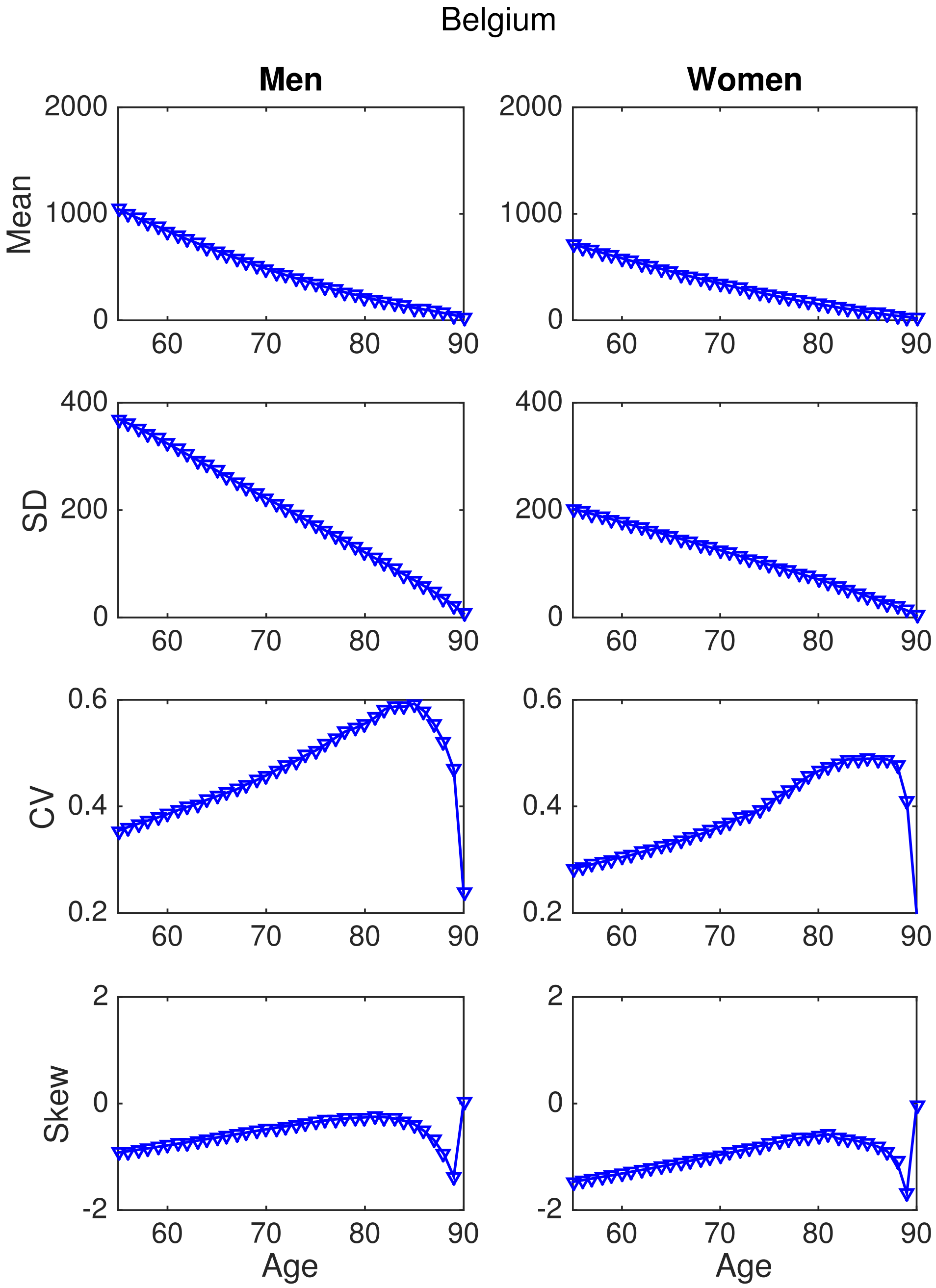

Figure B-1: Mean, standard deviation, coefficient of variation, and skewness for remaining healthy longevity (grip-years) as measured by grip strength, for Belgium

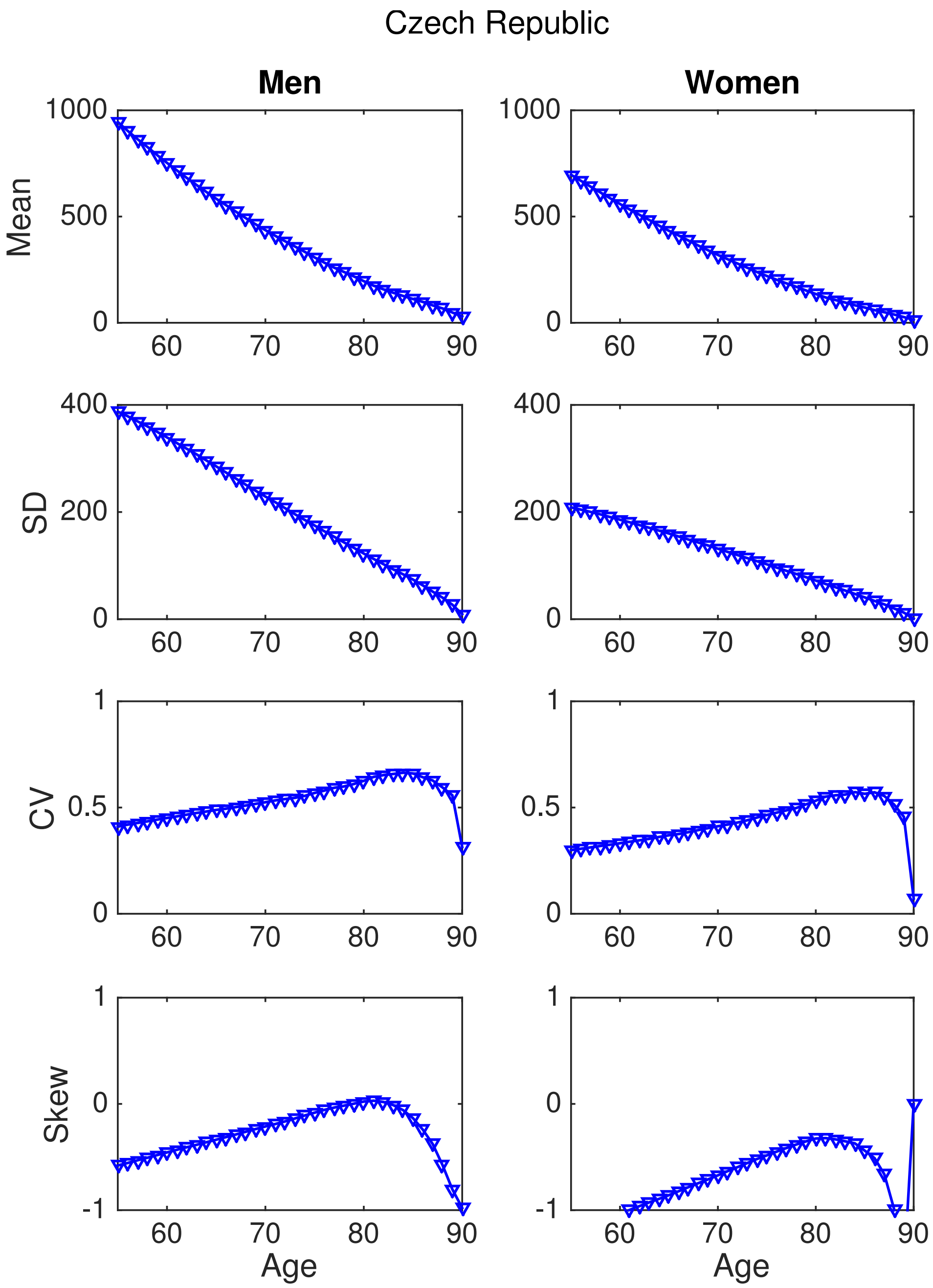

Figure B-2: As in Figure B-1, for Czech Republic

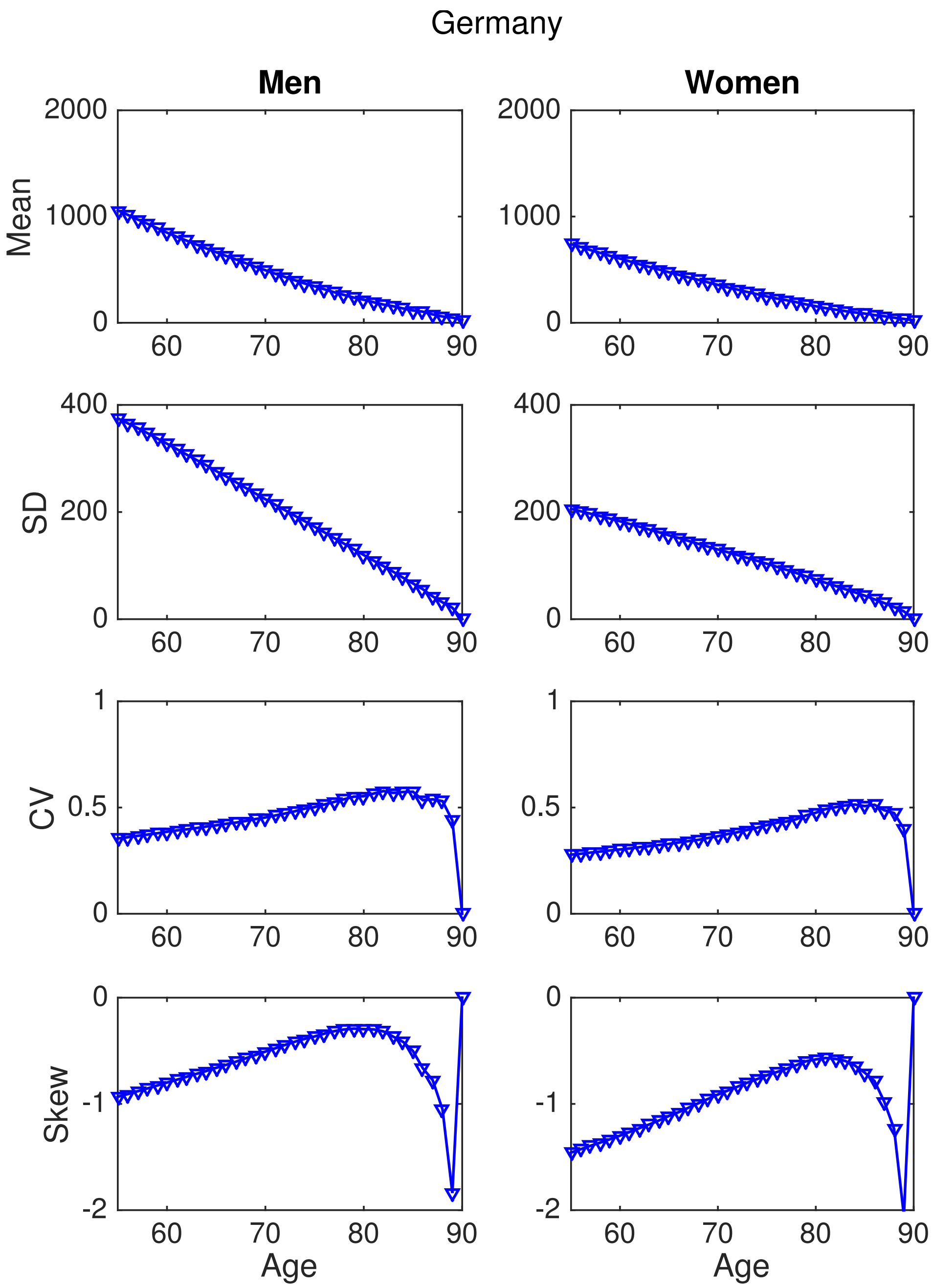

Figure B-3: As in Figure B-1, for Germany

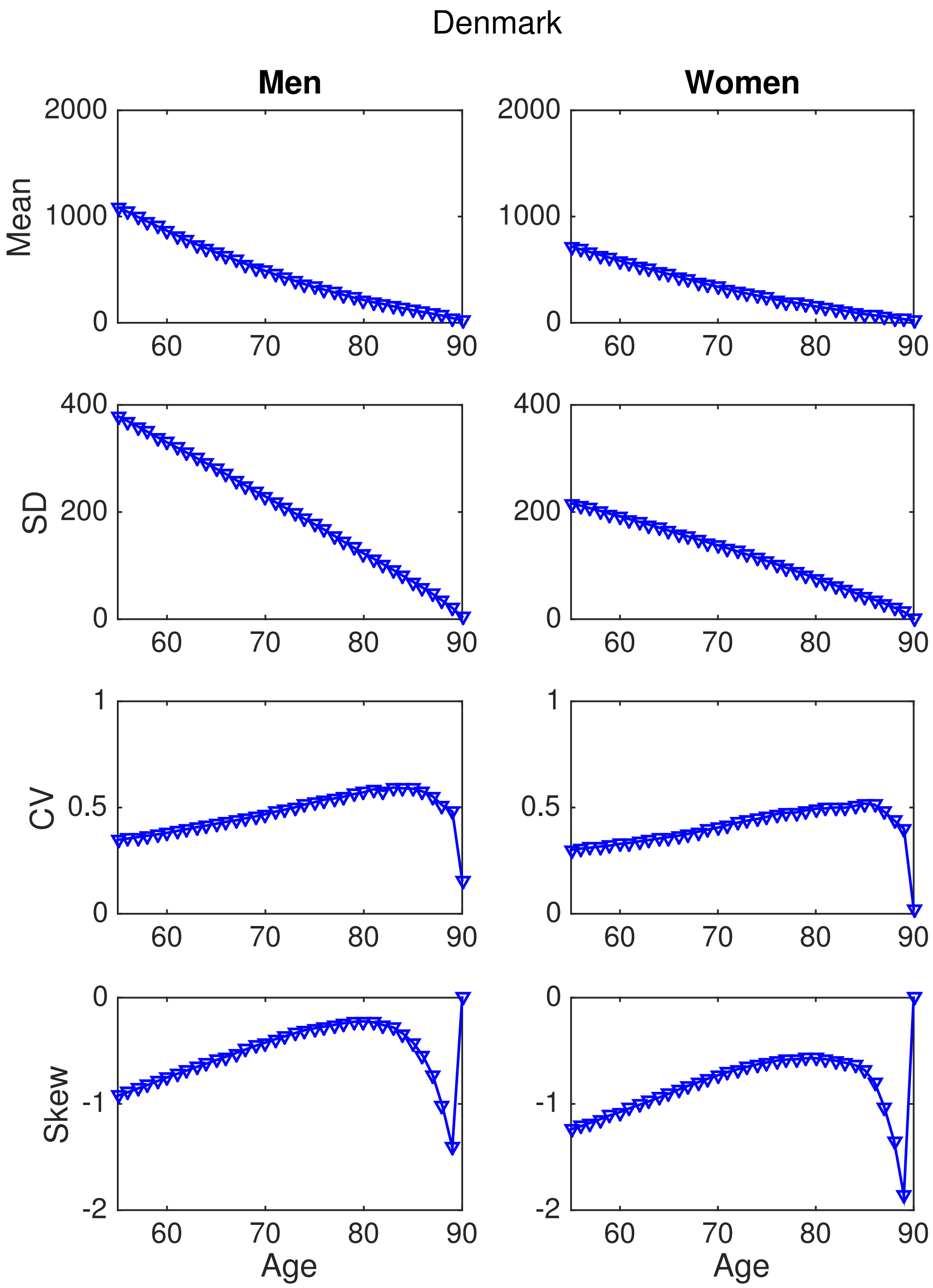

Figure B-4: As in Figure B-1, for Denmark

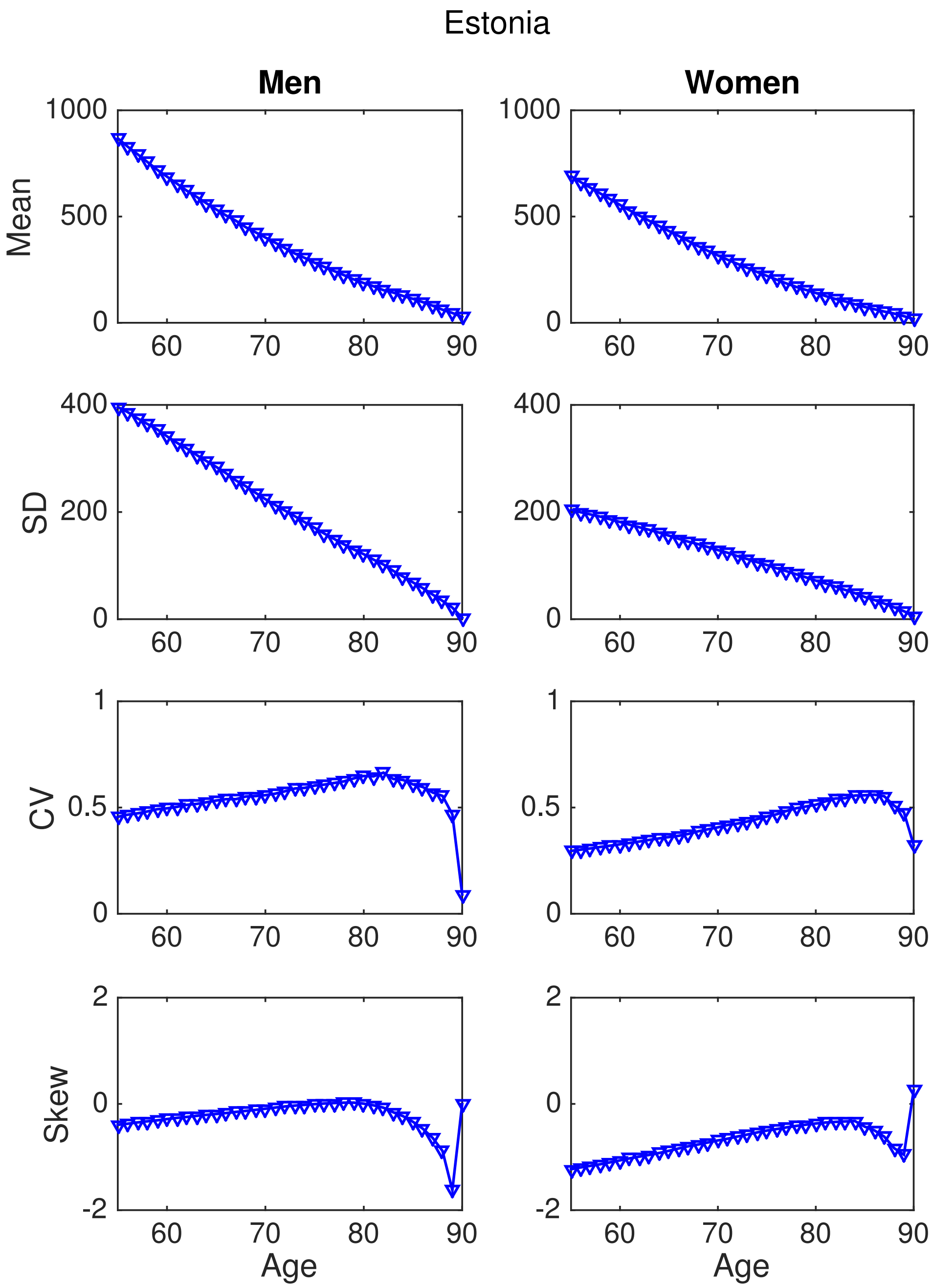

Figure B-5: As in Figure B-1, for Estonia

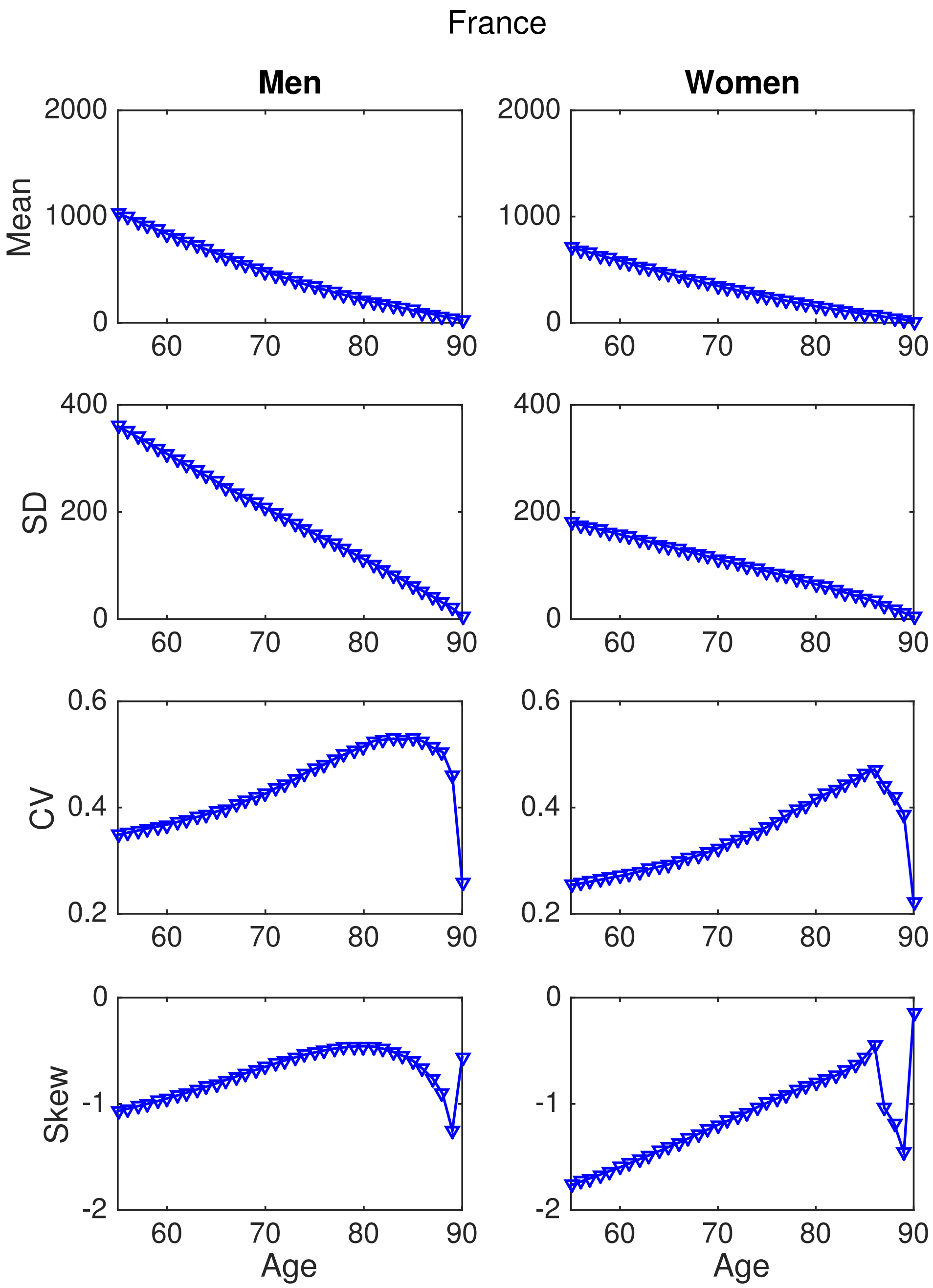

Figure B-6: As in Figure B-1, for France

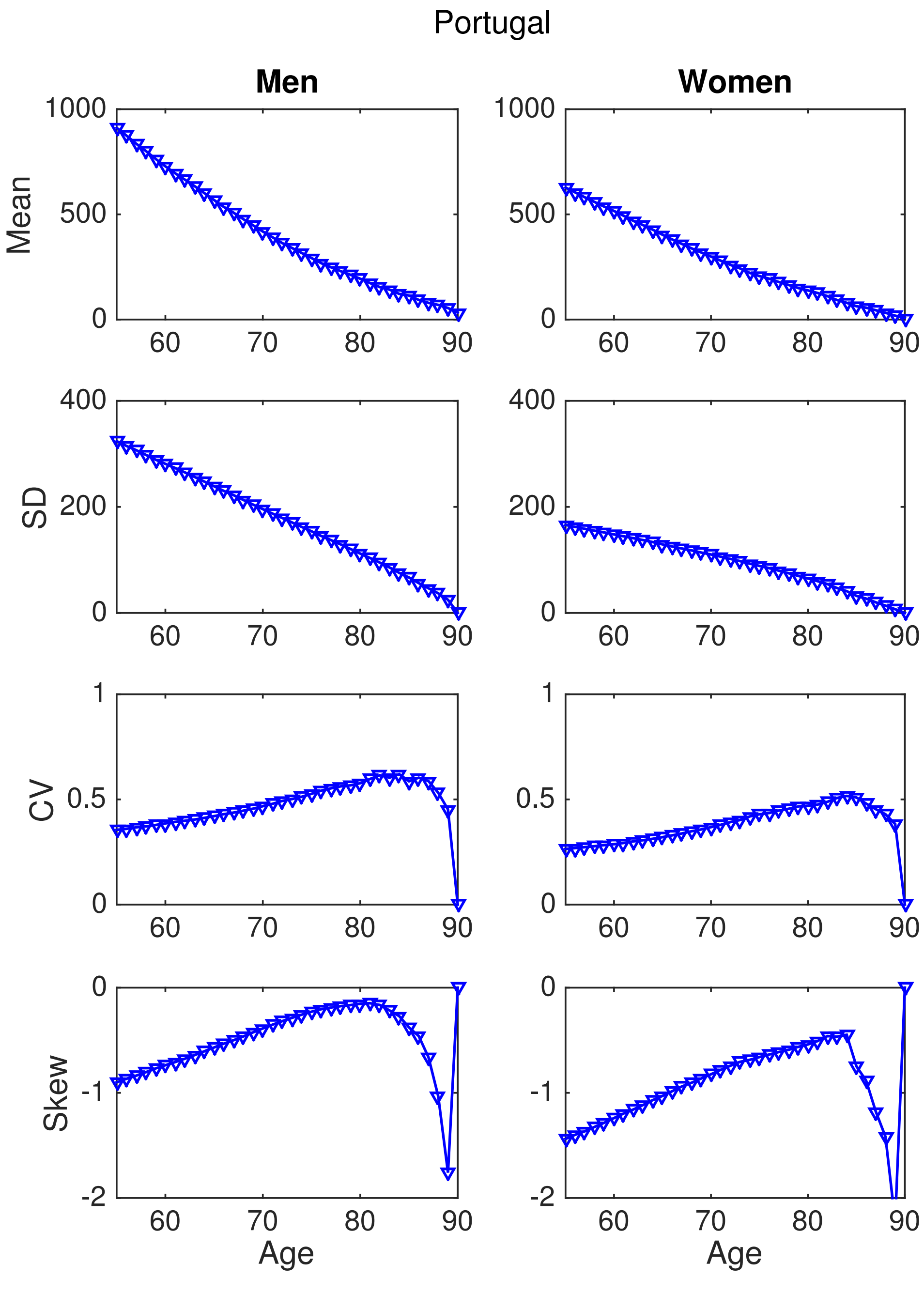

Figure B-7: As in Figure B-1, for Portugal

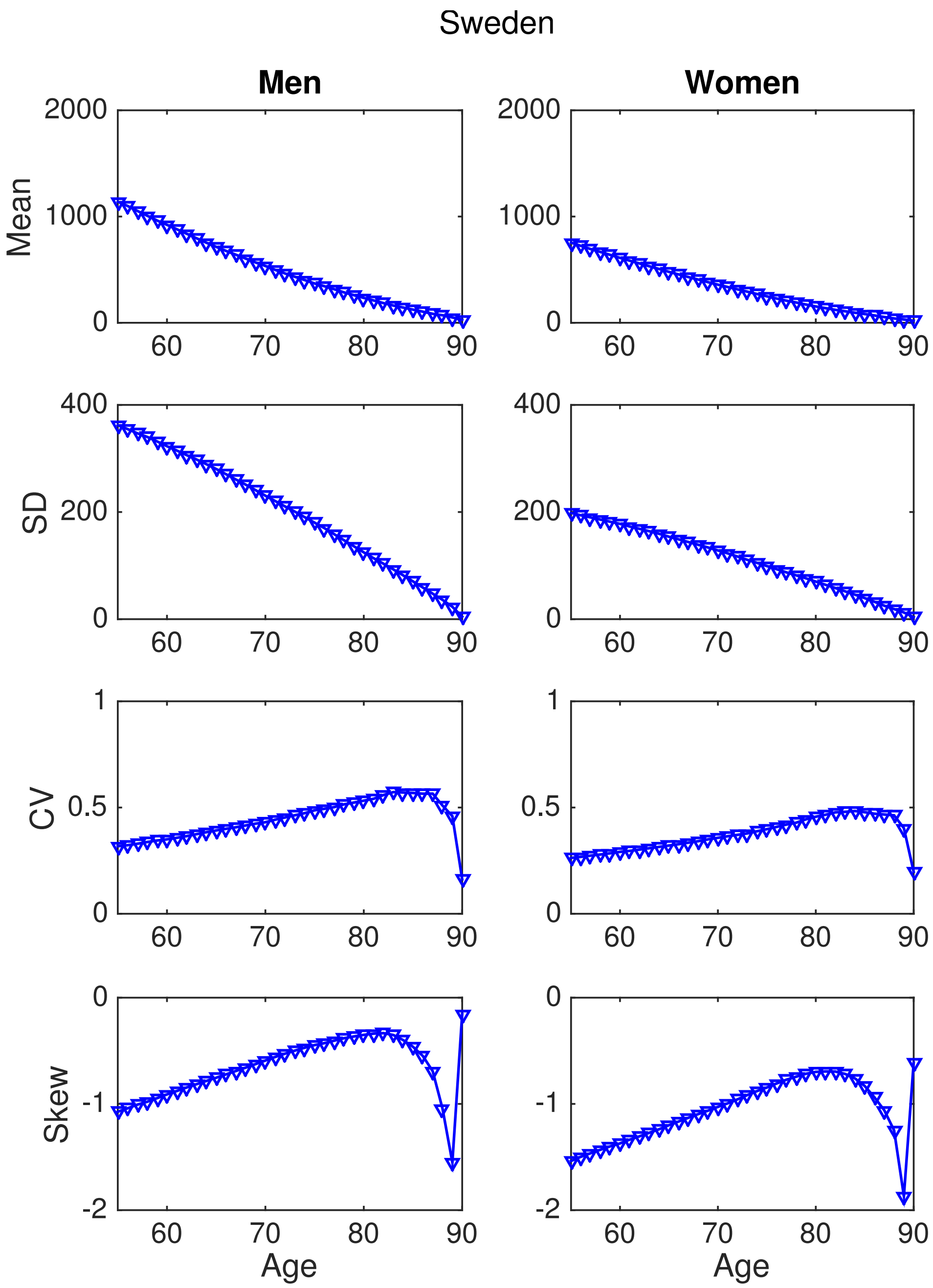

Figure B-8: As in Figure B-1, for Sweden

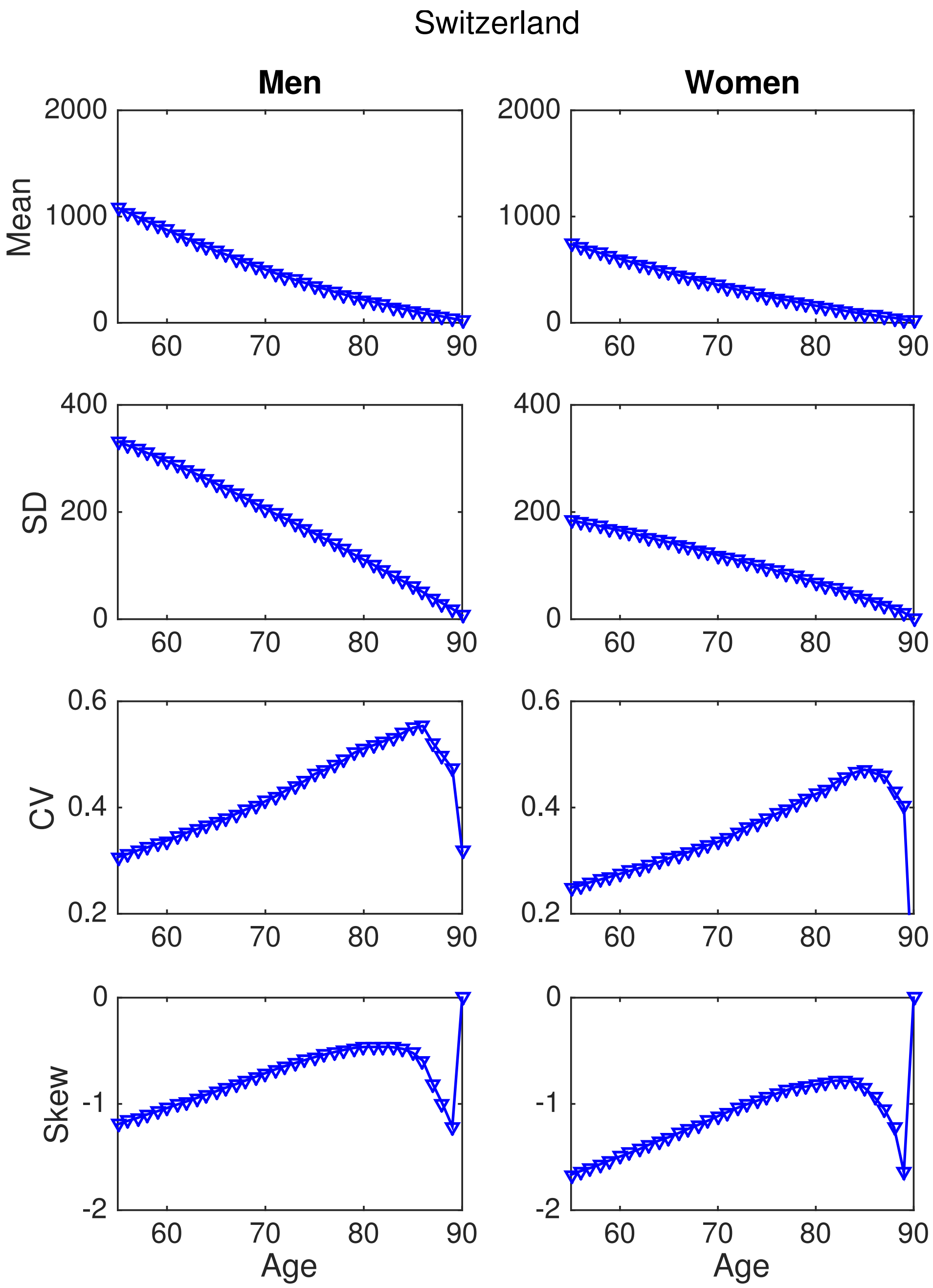

Figure B-9: As in Figure B-1, for Switzerland
